# The effect of me‐substituents of 1,4‐butanediol analogues on the thermal properties of biobased polyesters

**DOI:** 10.1002/pola.29074

**Published:** 2018-08-09

**Authors:** Frits van der Klis, Rutger J. I. Knoop, Johannes H. Bitter, Lambertus A. M. van den Broek

**Affiliations:** ^1^ Wageningen Food and Biobased Research, Bornse Weilanden 9, 6708 WG Wageningen The Netherlands; ^2^ Wageningen Biobased Chemistry and Technology, Bornse Weilanden 9, 6708 WG Wageningen The Netherlands

**Keywords:** biobased‐building blocks, glass transition, polyesters, structure–property relations

## Abstract

Biobased 1,4‐butanediol analogues are used to tune the glass transition temperature and crystallization in a series of polyesters and allow for the formation of stereocomplexes.

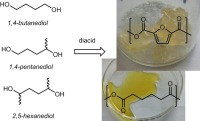

## INTRODUCTION

The use of biomass for the production of chemicals and materials can reduce our dependence on fossil fuels. It also opens opportunities to develop novel materials, which are not easily accessible from petrochemicals.

Starting from carbohydrates, one of the main constituents of biomass, both “novel” and “drop‐in” platform chemicals have been developed, of which some already reached commercialization.^1^, ^2^ Since carbohydrates are rich in oxygen functionality, conversion into oxygen rich building blocks, such as diacids and diols, offers great opportunities for the development of (novel) polyester materials.^3^ One example of a commercialized (bio‐degradable) polyester is poly(butylene succinate) (PBS),^4^ which can be 100% carbohydrate derived from the building blocks succinic acid^1^, ^3^, ^5^, ^6^ and 1,4‐butanediol.^1^, ^3^

The most explored route from carbohydrates to the PBS monomer 1,4‐butanediol is the reduction in C6‐sugar‐derived succinic acid^1^, ^3^; alternatively, hydrogenation of (C5‐sugar derived) furan under aqueous conditions could be used.^7^, ^8^ With the development of improved routes from C5‐ and C6‐sugars to furans, methyl‐substituted furans are becoming more available. For example, C5‐sugar‐derived furfural is selectively converted into both furan and 2‐methylfuran,^9^, ^10^ while C6‐sugar‐derived 5‐(hydroxymethyl)furan‐2‐carbaldehyde (HMF) is converted selectively into 2,5‐dimethylfuran.^11^ Similar to the conversion of furan to 1,4‐butanediol, aqueous hydrogenation of 2‐methylfuran gives 1,4‐pentanediol^12^, ^13^ and 2,5‐dimethylfuran gives 2,5‐hexanediol.^14^

**Figure 1:**
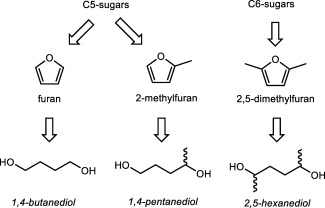
Sugar‐derived diol building blocks; 1,4‐butanediol‐analogues.

These three furan‐derived diols (Fig. 1) all share the same 1,4‐butanediol backbone, and could be considered as “methyl‐substituted 1,4‐butanediol‐analogues”: 1,4‐butanediol (0 × Me), 1,4‐pentanediol (1 × Me) and 2,5‐hexanediol (2 × Me). It is also clear that the introduction of methyl‐groups to 1,4‐butanediol introduces chiral centers.

These three butanediol analogues with varying numbers of methyl groups provide an interesting platform to prepare a series of derived (biobased) polyesters. To our knowledge, no systematic investigation on the influence of these methyl‐groups on polyester properties is reported.

It is, however, expected that these methyl‐groups should have a profound effect on the crystallization behavior and the glass transition temperature (*T*_g_) of derived polyesters, and thus could be used to tune polymer properties. This hypothesis is based on previously reported properties for succinate polyesters with 1,3‐propanediol analogues: 1,3‐propanediol (0 × Me): *T*_g_ = −44 °C, *T*_m_ = 45 °C;^15^ 2‐methyl‐1,3‐propanediol (1 × Me): *T*_g_ = −31 °C, *T*_m_ = absent/amorphous;^16^ and 2,2‐dimethyl‐1,3‐propanediol (2 × Me): *T*_g_ = −18 °C, *T*_m_ = 84 °C.^17^

So, for propanediol‐analogues, it has been shown that methyl groups have a significant influence on the properties of succinate polyesters made from them. Clearly, the orientation of the methyl groups plays an important role, since only the 2‐methyl‐1,3‐propanediol (irregular orientation of methyl groups after build‐in in the polymer chain) displays no apparent *T*_m_.

For the 1,4‐butanediols in our study, different enantiomers exist. It can be expected that optically pure derivatives of 2,5‐hexanediol (Fig. 1) provide a regular orientation of methyl‐groups, which might allow for crystallization of derived polyesters (as in the propanediol series, where regularity plays such an important role). These pure enantiomers can now be prepared via developed biochemical routes with excellent entiomeric excess, leading to the accessibility of optical pure (2*R*,5*R*)‐hexanediol^18^, ^19^, ^20^, ^21^ and (2*S*,5*S*)‐hexanediol.^18^ It is expected that the use of optically pure 2,5‐hexanediols should allow for crystallization due to the symmetry, and potentially the formation of the stereocomplex when (2*R*,5*R*)‐hexanediol‐ and (2*S*,5*S*)‐hexanediol‐derived polyesters are mixed in a 1:1 ratio.

**Figure 2:**
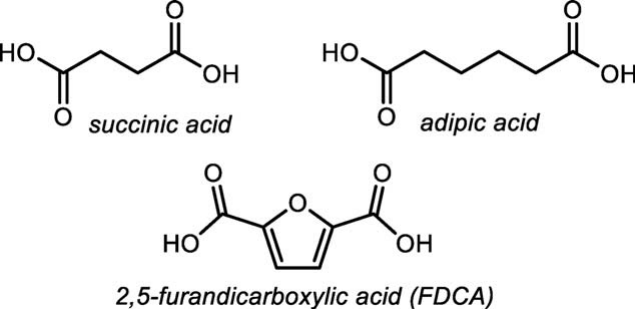
Sugar‐derived diacid building blocks.

Here, we report on the systematic investigation of the influence of Me‐groups on 1,4‐butanediol‐analogues in three series of polyesters. For these three series, a combination of the diols with (potentially) carbohydrate‐derived diacids was chosen: succinic acid, adipic acid,^22^, ^23^, ^24^ and 2,5‐furandicarboxylic acid^25^, ^26^ (Fig. 2).

The influence of methyl‐groups on 1,4‐butanediol‐analogues on the thermal properties was first investigated for the most flexible and aliphatic diacid building block, that is, adipic acid.

Poly(butylene adipate) is already a well‐studied polyester,^27^ and was therefore not prepared, but for comparison, the literature data on poly(butylene adipate) are shown in Table 1, entry 1. Poly(butylene adipate) is a polyester with a low *T*_g_ (*T*_g_ = −68/−60 °C) but, due to its linear structure, it is still able to crystalize, and therefore has a melting temperature of 54–60 °C (Table 1, entry 1). Next, 1,4‐pentanediol (racemic) and 2,5‐hexanediol (racemic) were prepared starting from 2‐methylfuran and 2,5‐dimethylfuran (see supporting information), and the corresponding adipate polyesters were prepared. Polymerizations were performed at small scale (1.5 g diol), by transesterification of dimethyl‐adipate with the diols (30 mol % excess diol relative to diester). Both the materials were obtained as sticky yellow oils, and the materials were characterized on molecular weight (GPC) and thermal properties (DSC and TGA). The results of the measurements are summarized in Table 1, entries 2–3.

**Table 1:** Properties of Melt Polymerization Products, Obtained Molecular Weights, Thermal Properties, and Appearance

Entry	Diol^a^	Di‐acid^a^	*M*_n_ b (10^3^ g mol^−1^)	*M*_w_ b (10^3^ g mol^−1^)	PDI^b^	*T*_g_ c (°C)	*T*_m_ c (°C)	*T*_d5_ d (°C)	Appearance
1	1,4‐BDO	Adipic	−	−	−	−68/−60	54–60	−	Literature^27^
2	1,4‐PDO	Adipic	4.2	19.1	4.6	−52	–	265	Transp. yellow sticky
3	2,5‐HDO	Adipic	5.5	19.6	3.6	−39	−	296	Transp. yellow sticky
4	1,4‐BDO	Succinic	–	–	–	−33	114–115	–	Literature^27^
5	1,4‐PDO	Succinic	8.3	15.8	1.9	−29	−	275	Transp. light yellow sticky
6	2,5‐HDO	Succinic	3.7	7.1	1.9	−14	–	281	Transp. colorless sticky
7^e^	2*R*,5*R*‐HDO	Succinic	7.8	17.8	2.3	−19	−	256	Transp. colorless sticky
8	2*S*,5*S*‐HDO	Succinic	3.8	7.1	1.9	−18	200	264	Transp. colorless / opaque white sticky
9	SC‐HDO	Succinic	−	−	−	−17	−	−	Opaque white sticky
10	1,4‐BDO	2,5‐FDCA	11.8–21.4	21.2–55.4	1.8–2.5	36–38	170–177	304	Literature^28^, ^29^
11	1,4‐PDO	2,5‐FDCA	8.1	29.5	3.6	47	−	248	Transp. yellow, brittle
12	2,5‐HDO	2,5‐FDCA	5.7	13.6	2.4	51	–	250	Transp. yellow, hard
13	2*R*,5*R*‐HDO	2,5‐FDCA	4.5	11.0	2.4	52	−	−	Semi‐transp. light green, hard
14	2*S*,5*S*‐HDO	2,5‐FDCA	4.9	9.3	1.9	52	–	248	Semi‐transp. light green, hard

a 1,4‐BDO = 1,4‐butanediol, 1,4‐PDO = 1,4‐pentanediol (racemic mixture), 2,5‐HDO = 2,5‐hexanediol (racemic mixture), SC‐HDO = stereo complex of 1:1 ratio 2*R*,5*R*‐HDO and 2*S*,5*S*‐HDO polyester, 2,5‐FDCA = furan‐2,5‐dicarboxylic acid.

b GPC and DSC measurements were performed on the crude products.

c *T*_g_ and *T*_m_ are obtained from the second DSC heating runs recorded at 10 °C min^−1^.

d 5 wt % loss temperature measured on a TGA, at a heating rate of 10 °C min^−1^ in N_2_.

The increased *M*_w_ compared to the other succinate analogues (entries 6 and 8) can be explained by a prolonged reaction time at 210 °C (2h instead of 1h), see ESI..

When an additional methyl‐group into the 1,4‐butanediol structure was introduced, thus poly(1,4‐pentylene adipate), an increase in the *T*_g_ (−52 °C) and a loss of crystallinity was observed, as apparent from the absence of a *T*_m_ (Table 1, entry 2). Addition of two methyl‐groups, thus poly(2,5‐hexylene adipate), further increases the *T*_g_ to −39 °C, and still no apparent *T*_m_ was observed (Table 1, entry 3). These results indicate that the introduction of methyl‐groups increases the *T*_g_, and that the racemic diols disturb the crystallization of the adipate‐polyesters, making them fully amorphous.

Next, a shorter chain building block was investigated, that is, succinic acid (slightly less flexible and less aliphatic compared with adipic acid). As mentioned above, PBS is a commercial biodegradable polyester, with a *T*_g_ of −33 °C and a melting temperature of 114–115 °C (Table 1, entry 4). The racemic methyl‐analogues, poly(1,4‐pentylene succinate) and poly(2,5‐hexylene succinate), were prepared and the properties are listed in Table 1, entries 5–6. Introduction of 1 methyl‐group, poly(1,4‐pentylene succinate), increases the *T*_g_ from −33 to −29 °C, and resulted in loss of *T*_m_ (Table 1, entry 5). Addition of a second methyl‐group, poly(2,5‐hexylene succinate), further increases the *T*_g_ to −14 °C, and still no apparent *T*_m_ was observed (Table 1, entry 6). The results of the succinate‐series are thus comparable to the results obtained in the adipate‐series.

It was decided to investigate the effect of the stereo conformation of 2,5‐hexanediols on the crystallinity of succinate polyesters. It was expected that for the succinate polyesters, which are slightly less flexible and less aliphatic compared with adipic acid, an effect should be visible. Therefore, the two optically pure 2,5‐hexanediol‐succinates, that is, poly(2*R*,5*R*‐hexylene succinate) and poly(2*S*,5*S*‐hexylene succinate), were prepared (Table 1, entries 7–8). Initially, both materials were transparent directly after polymerization, with a *T*_g_ of −18 to −19 °C, and no apparent *T*_m_ (Table 1, entries 7–8). When the samples were stored at 50 °C to induce crystallization, indeed one of the polymers (poly(2*S*,5*S*‐hexylene succinate); Table 1, entry 8) turned opaque, indicating crystal formation. DSC analysis showed a *T*_m_ of 200 °C, confirming the visual observation. However, the crystallization was very slow, since it took 3 months before the sample turned opaque.

Next, the possibility of stereo‐complex formation was investigated, to obtain SC‐HDO‐succinate. A 1:1 mixture of poly(2*S*,5*S*‐hexylene succinate) and poly(2*R*,5*R*‐hexylene succinate) was dissolved in chloroform, and the solvent was allowed to slowly evaporate. When the sample was dry, immediately an opaque structure was visible, indicating crystallinity. The material was still sticky, indicating a low degree of crystallinity. DSC analysis up to 200 °C did not show a *T*_m_, and therefore could not confirm the formation of the stereo‐complex (Table 1, entry 9). However, the *T*_m_ of the stereocomplex is expected to be higher compared with the *T*_m_ (200 °C) of the poly(2*S*,5*S*‐hexylene succinate). Also the speed of crystallization was much faster, which are both in accordance with the theory on stereo complex formation (higher *T*_m_ and faster crystallization).^30^ However, the opaque color of the sample is a very strong indication that the stereocomplex has been formed. The *T*_g_ of the stereo‐complex was −16 °C, which is in the same range as the parent 2*R*,5*R‐* and 2*S*,5*S*‐(hexylene succinate)polyesters (*T*_g_ of −19 to −18 °C) (Table 1, entries 7–9).

Next, polyesters based on a more rigid bio‐building block were prepared: furan‐2,5‐dicarboxylic acid (FDCA). The polyester product of 1,4‐butanediol and FDCA, poly(butylene furanoate) (PBF) was previously synthesized, and the properties are listed in Table 1, entry 10. PBF has a *T*_g_ of 36–38 °C, and a melting temperature of 170–177 °C. Next, the racemic methyl‐analogues, poly(1,4‐pentylene furanoate) and poly(2,5‐hexylene furanoate), were prepared; the properties of which are listed in Table 1, entries 11–12. The optical pure hexanediol polyesters, poly(2*S*,5*S*‐hexylene furanoate) and poly(2*R*,5*R*‐hexylene furanoate), were also prepared (Table 1, entries 13–14).

Introduction of one methyl‐group (poly(1,4‐pentylene furanoate) increases the *T*_g_ from 37 to 47 °C, and resulted in loss of *T*_m_ (Table 1, entries 10–11). Addition of a second methyl‐group, poly(2,5‐hexylene furanoate), further increases the *T*_g_ to 51 °C, and still no apparent *T*_m_ was observed (Table 1, entry 12). The optical pure analogues have the same thermal properties as the racemic hexanedol‐analogue, as expected (Table 1, entries 13–14).

**Figure 3:**
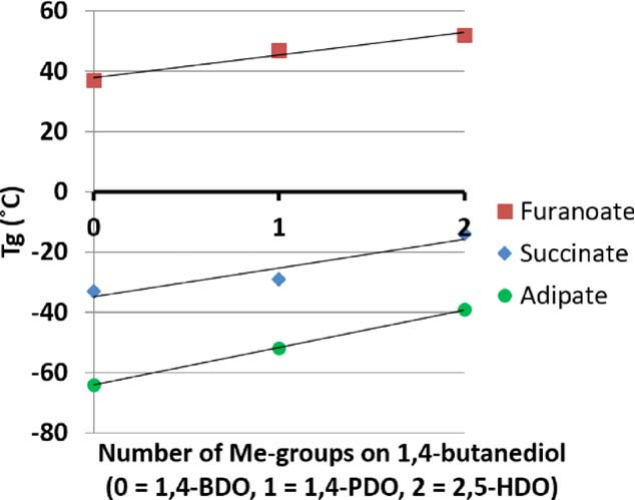
*T*_g_ of polyesters from 1,4‐butanediol‐analogues, as a function of the number of methyl‐groups (0 = 1,4‐BDO; 1 = 1,4‐PDO; 2 = 2,5‐HDO). [Color figure can be viewed at http://wileyonlinelibrary.com]

The *T*_g_s of the three polyester series (adipate, succinate, and furanoate) were plotted as a function of the number of methyl‐groups on 1,4‐butanediol; 0 × Me (1,4‐butanediol), 1 × Me (1,4‐pentanediol) and 2 × Me (2,5‐hexanediol). The results are shown in Figure 3.

All three series showed an increase in the *T*_g_ with increasing numbers of methyl‐groups. Surprisingly, the slopes of all series (furanote‐, succinate‐, and adipate‐) display the same trend in a parallel direction. This indicates that the found correlation (increasing *T*_g_ with increasing number of Me‐groups) might be a universal feature, and that this knowledge could be used to predict *T*_g_s for other polyester series. In addition, the same feature is reported for polyesters with 1,3‐propanediol analogues, where an an increase in Me‐groups resulted in an increase in *T*_g_.^15^, ^16^, ^17^

To summarize, a systematic investigation on the influence of methyl‐groups of 1,4‐butanediol‐analogues of adipate‐, succinate‐, and furanoate‐polyesters was investigated. All series showed an increase in *T*_g_ with an increasing number of methyl‐groups. The chirality of these methyl‐groups was also found to influence the crystallization behavior. For the first time, we have a strong indication that a stereocomplex of poly(2*R*,5*R*‐hexylene succinate) and poly(2*S*,5*S*‐hexylene succinate) can be formed (visual observation of crystallization). The use of these carbohydrate based 1,4‐butanediol‐analogues provides a nice tool for further tuning of other (co)‐polyesters.

## EXPERIMENTAL

A description of the materials, analytical equipment, and experimental methods can be found in the Supporting Information.

## Supporting information

Supporting Information
