# Supplementary material for: The effect of me‐substituents of 1,4‐butanediol analogues on the thermal properties of biobased polyesters
Source: J Polym Sci A Polym Chem. 2018 Aug 9;56(17):1903–6. doi: 10.1002/pola.29074 (PMC6174968; doi:10.1002/pola.29074)
Supplement: Supplementary file 1 — Supporting Information [file POLA-56-1903-s001.pdf]

Supporting Information

The effect of Me-substituents of 1,4-butanediol analogues on the thermal properties of biobased polyesters

Frits van der Klis, Rutger J. I. Knoop, Johannes H. Bitter,

*Lambertus A. M. van den Broek**

***Corresponding author;** E-mail: ben.vandenbroek@wur.nl

Contents

1. Materials
2. Analytical equipment
3. Synthesis of substituted butanediols
   3.1 Synthesis of 1,4-pentanediol (mixture of isomers)
   3.2 Synthesis of 2,5-hexanediol (mixture of isomers)
4. Melt polymerizations
5. Polyester properties
   5.1 Thermogravimetric analysis (TGA)
   5.2 Differential scanning calorimetry (DSC)
   5.3 Matrix assisted laser desorption time-of-flight mass spectrometry (MALDI TOF-MS)
6. References

1. Materials

The following chemicals and solvents were used without further purification: 1,4-butanediol (ReagentPlus, 99%, Sigma Aldrich), Celite 545 (filter aid, treated with sodium carbonate, flux calcined, Sigma Aldrich), dimethyl-adipate (>99%, Sigma Aldrich), 2,5-dimethylfuran (99%, Sigma Aldrich), dimethyl-succinate (Provichem 2511, gift from Proviron), 2R,5R-hexanediol (>99.0%, sum of enantiomers, GC, Aldrich), 2S,5S-(+)-hexanediol (99%, 99% ee, Acros Organics), hydrogen (5.0, Linde Gas Benelux B.V.), 2-methylfuran (>98%, Sigma Aldrich), nitrogen (4.5, Linde Gas Benelux B.V.), O-xylene (puriss. p.a., >99.0%, GC, Sigma Aldrich), ruthenium, 5% on activated carbon (50-70% wetted powder, Evonik Noblyst P3060, 5.0% Ru, 65.8 wt% H_2O, STREM), titanium(IV)isopropoxide (97%, Aldrich), toluene (for analysis, Merck). Dimethyl-FDCA was prepared and re-crystallized as reported earlier.[1]

2. Analytical equipment

GC-MS analyses and NMR measurements were performed as described earlier.[2] Gel permeation chromatography (GPC), and differential scanning calorimetry (DSC) was performed as described previously.[1]
Thermogravimetric analysis (TGA) was measured with a Perkin Elmer STA 6000 (simultaneous thermal analyzer). In a ceramic cup 10-15 mg sample was weighed and heated from 30 to 600°C with 10°C/min under a flow of nitrogen (30 mL/min). The results (weight loss) were evaluated with Perkin Elmer Pyris software.
Matrix assisted laser desorption time-of-flight mass spectrometry (MALDI TOF-MS) was performed with a Bruker UltraFlextreme (Bruker Daltonics, Bremen, Germany) in reflective mode and positive ions were examined. Samples and standards were prepared according to Cruz-Izquierdo *et al.*[3] Ten spectra with a total of 50 shoots per spot were collected using the lowest possible laser intensity that led to a good quality spectrum.

3. Synthesis of substituted butanediols

3.1 Synthesis of 1,4-pentanediol (mixture of isomers)

A 600 mL Parr pressure reactor was charged with water (100 g, 5.55 mol), 2-methylfuran (100 g, 1.22 mol) and 5wt% Ru/C (10 g wetted powder, 1.69 mmol Ru). The reactor was closed and flushed three times with 4 bar N_2. Hydrogen (80 bar) was applied, and the reactor was heated to 120 ˚C under

stirring (300 rpm). *Please note that the reaction is exothermic!* The reaction was indicated by a fast drop in hydrogen pressure, and additional hydrogen was added regularly to maintain a pressure of 80 bar. After 6h at 120 ˚C, the consumption of hydrogen stopped, and the reactor was allowed to cool to room temperature. The reactor was flushed with N_2, and opened. The catalyst was removed by filtration of the reaction mixture over Celite (Buchner funnel type 3). The Celite was washed with water, and the combined slightly brown aqueous phase was concentrated on a rotary evaporator (50 ˚C) to give 92.8 g light brown oil. Residual water was removed by a Dean-Stark set-up with toluene (~200 mL) for 19h, followed by removal of the toluene on a rotary evaporator (40 ˚C), to give 91.4 g light brown oil. Purification by vacuum distillation (heating mantle 120 ˚C, distillation temp. 80-100 ˚C, 1-0.2 mbar), gave the desired 1,4-pentanediol (mixture of isomers, 84.9 g, yield 82 mol%) as a viscous colourless oil.

^{1}H NMR (400.17 MHz, $CDCl_3$, δ): 4.48 (s, 1H; OH), 4.31 (s, 1H; OH), 3.84-3.67 (m, 1H; CH), 3.65-3.48 (m, 2H; CH_2), 1.71-1.55 (m, 2H; CH_2), 1.55-1.35 (m, 2H; CH_2), 1.13 (d, *J* = 6.3 Hz, 3H; CH_3); ^{13}C NMR (100.62 MHz, $CDCl_3$, δ): 67.4 (C4), 62.2 (C1), 35.8 (C3), 28.8 (C2), 23.1 (C5);[4] EIMS (*m/z* (%)): 89 (3) [$C_4H_{11}O_2^+$], 85 (3) [$C_5H_9O^+$], 71 (100) [$C_4H_8O^+$], 56 (17) [C_4H_8], 45 (96) [$C_2H_5O^+$], 42 (70) [C_3H_6].

3.2 Synthesis of 2,5-hexanediol (mixture of isomers)

A 600 mL Parr pressure reactor was charged with water (100 g, 5.55 mol), 2,5-dimethylfuran (100 g, 1.22 mol) and 5wt% Ru/C (8.5g wetted powder, 1.44 mmol Ru). The reactor was closed and flushed three times with 4 bar N_2. Hydrogen (80 bar) was applied, and the reactor was heated to 120 ˚C under stirring (300 rpm). *Please note that the reaction is exothermic!* The reaction was indicated by a fast drop in hydrogen pressure, and additional hydrogen was added regularly to maintain a pressure of 80 bar. After 4h at 120 ˚C, the consumption of hydrogen stopped, and the reactor was allowed to cool to room temperature. The reactor was flushed with N_2, and opened. The catalyst was removed by filtration of the reaction mixture over Celite (Buchner funnel type 3). The Celite was washed with water, and the combined slightly hazy/brown aqueous phase was concentrated on a rotary evaporator (50 ˚C) to give 100 g light brown hazy oil. Residual water was removed by a Dean-Stark set-up with toluene (~200 mL) for 19h, followed by removal of the toluene on a rotary evaporator, to give 76.9 g slightly coloured hazy liquid with brown particles. Purification by vacuum distillation (heating mantle 120 ˚C, distillation temp. 80-100 ˚C, 1-0.2 mbar), gave the desired 2,5-hexanediol (mixture of isomers, 70.27 g, yield 57 mol%) as a viscous colourless oil.

^{1}H NMR (400.17 MHz, $CDCl_3$, δ): 3.84-3.65 (m, 4H; OH & CH), 1.48 (m, 4H; CH_2), 1.12 (d, *J* = 6 Hz, 6H; CH_3); ^{13}C NMR (100 MHz, $CDCl_3$, δ): 68.3 (*anti*-C2/C5), 67.6 (*syn*-C2/C5), 36.1 (*anti*-C3/C4), 34.9 (*syn*-C3/C4), 23.7 (*anti*-C1/C6), 23.3 (*syn*-C1/C6);[5] EIMS (*m/z* (%)): 85 (53) [$C_5H_9O^+$], 83 (3) [$C_5H_7O^+$], 67 (17) [$C_5H_7^+$], 56 (100) [C_4H_8], 45 (83) [$C_2H_5O^+$], 41 (53) [$C_3H_5^+$].

4. Melt polymerizations

All melt polymerizations were performed according to the following standardized protocol: A 100 mL 3-neck bottle was equipped with a magnetic-drive mechanical stirrer, and a Claisen distillation set-up. The bottle was charged with dimethyl-ester and diol (0.5-1.5 g diol, 30% molar excess relative to dimethyl ester). (For all reactions with dimethyl-FDCA, 4 mL o-xylene was added to prevent sublimation). Three cycles of vacuum/nitrogen were applied, and the mixture was heated under stirring to 115 ˚C. After a homogeneous mixture was obtained, titanium(IV)isopropoxide (1.25 mol%) was added under a flow of nitrogen. To facilitate the formation of oligomers, the temperature was increased to 145 ˚C and kept at this temperature for 1h. Next, the temperature was stepwise increased to 170 ˚C (3h), 190 ˚C (1h), and 210 ˚C (1h). To further increase the molecular weight, and remove the excess of diol, at 210 ˚C vacuum was applied (10 – 0.1 mbar) for about 1h, or until the mixture became too viscous for continuously stirring. The bottle was allowed to cool to room temperature, and the polymer was collected.

[*Note:* The standard protocol described above was used for all polyesters. However, the 2*R*,5*R*-HDO / succinate polyester from Table 1, entry 7 (main article), was kept at 210 ˚C for 2h instead of 1h during synthesis. Which also explains the higher Mw compared to the other polyesters in the same succinate series (Table 1, entries 6 and 8).]

5. Polyester properties

5.1 Thermogravimetric analysis (TGA)

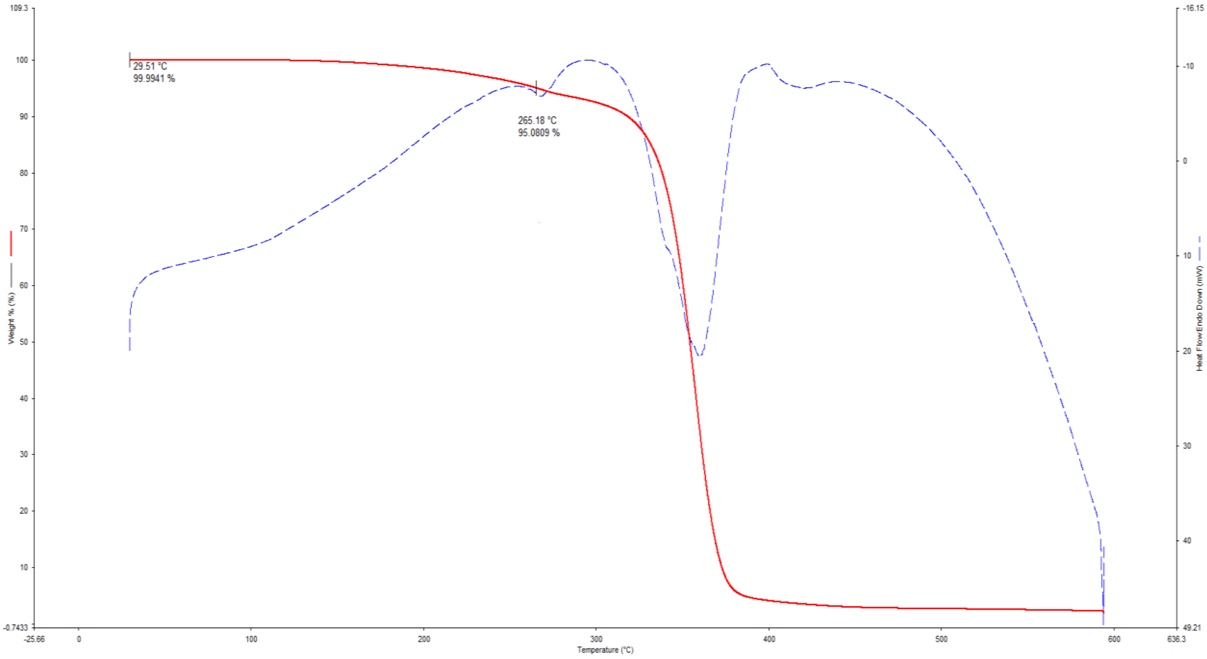

FIGURE S1: TGA of 2; Poly(1,4-pentylene adipate)

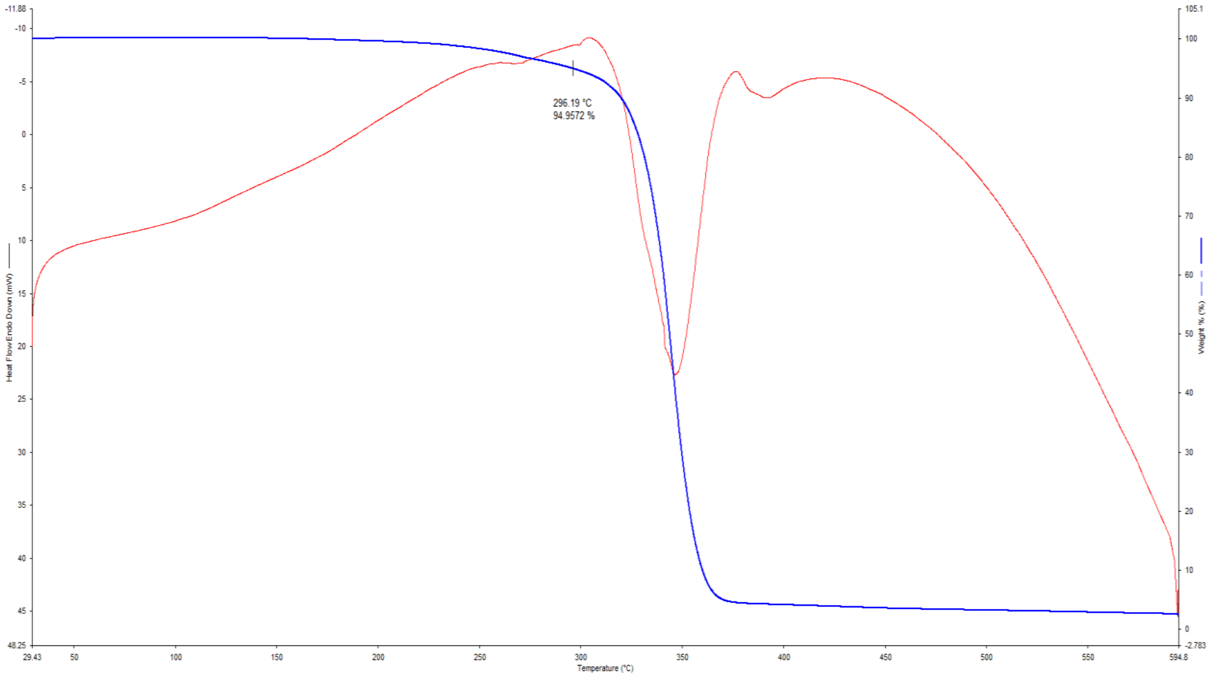

FIGURE S2: TGA of 3; Poly(2,5-hexylene adipate)

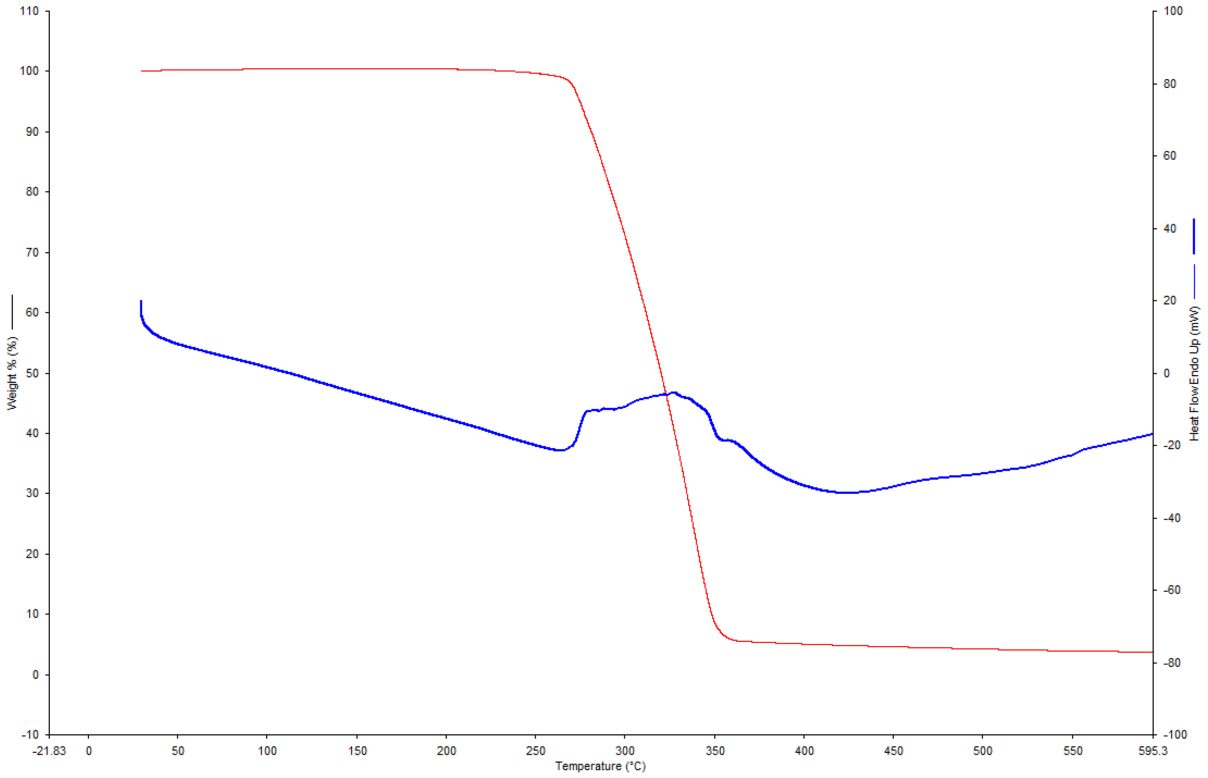

FIGURE S3: TGA of 5; Poly(1,4-pentylene succinate)

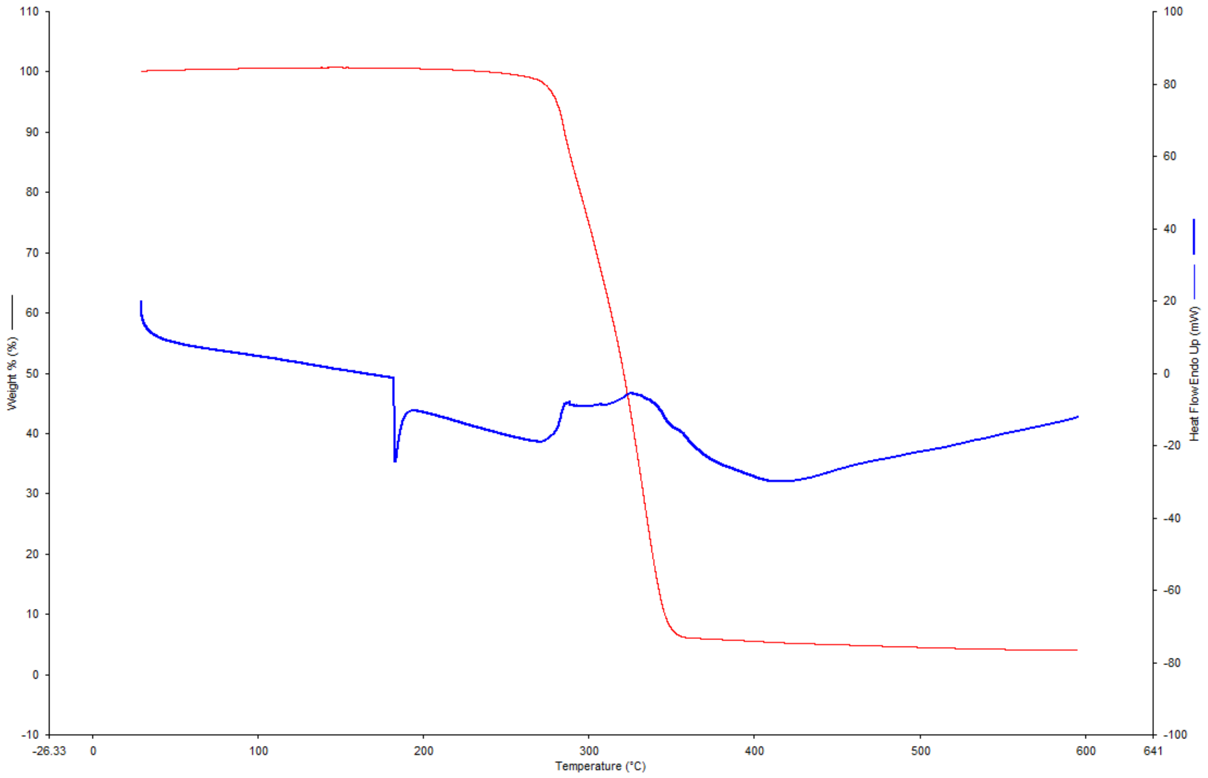

FIGURE S4: TGA of 6; Poly(2,5-hexylene succinate)

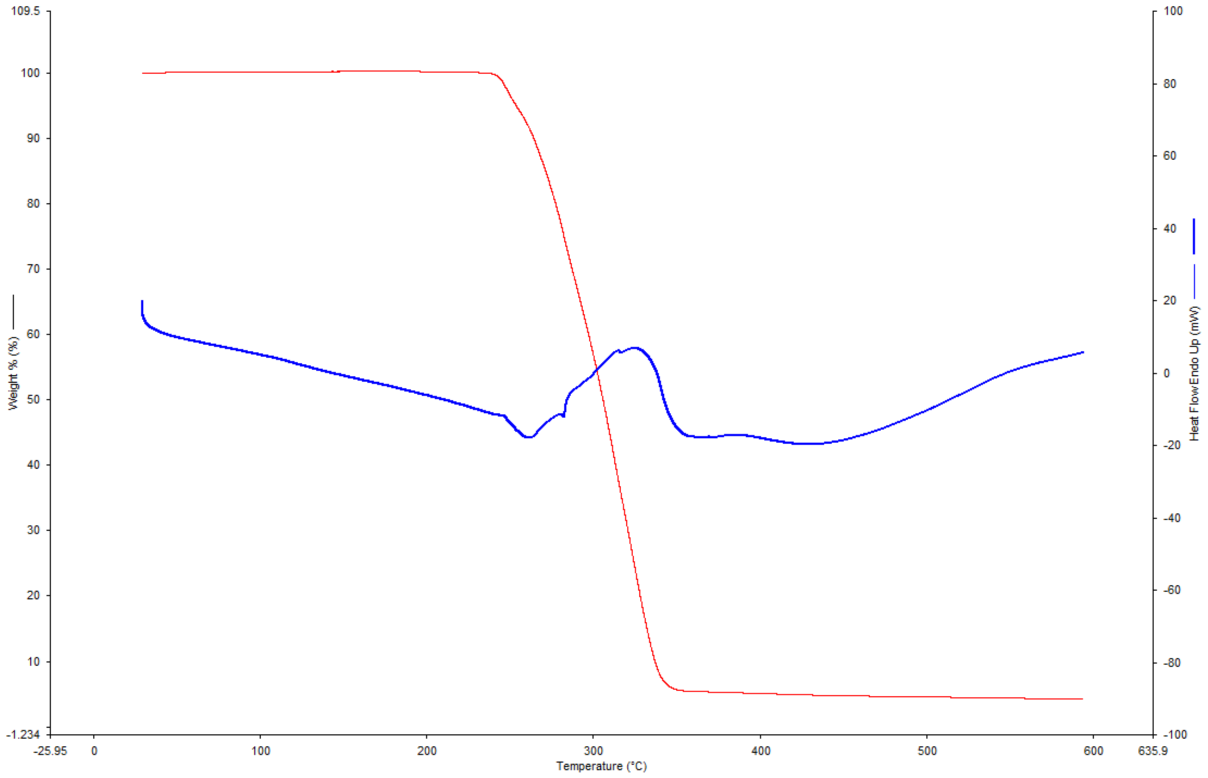

FIGURE S5: TGA of 7; Poly(2*R*,5*R*-hexylene succinate)

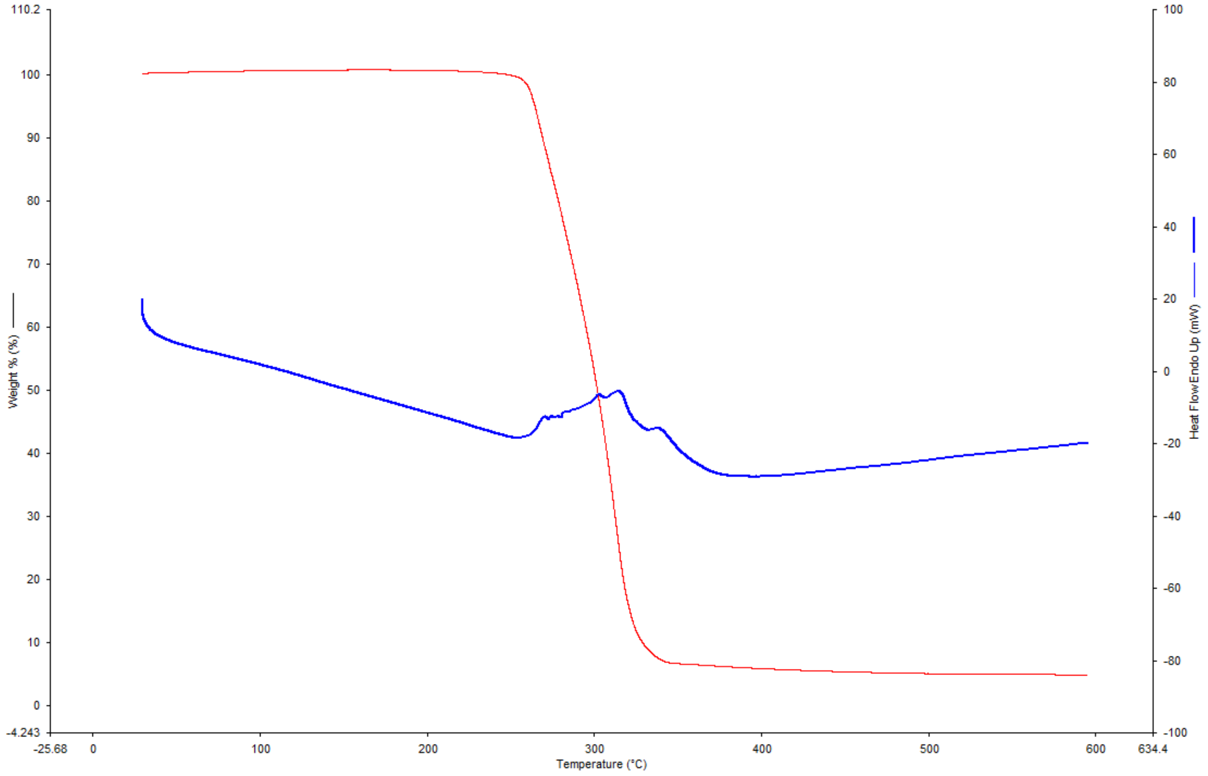

FIGURE S6: TGA of 8; Poly(2*S*,5*S*-hexylene succinate)

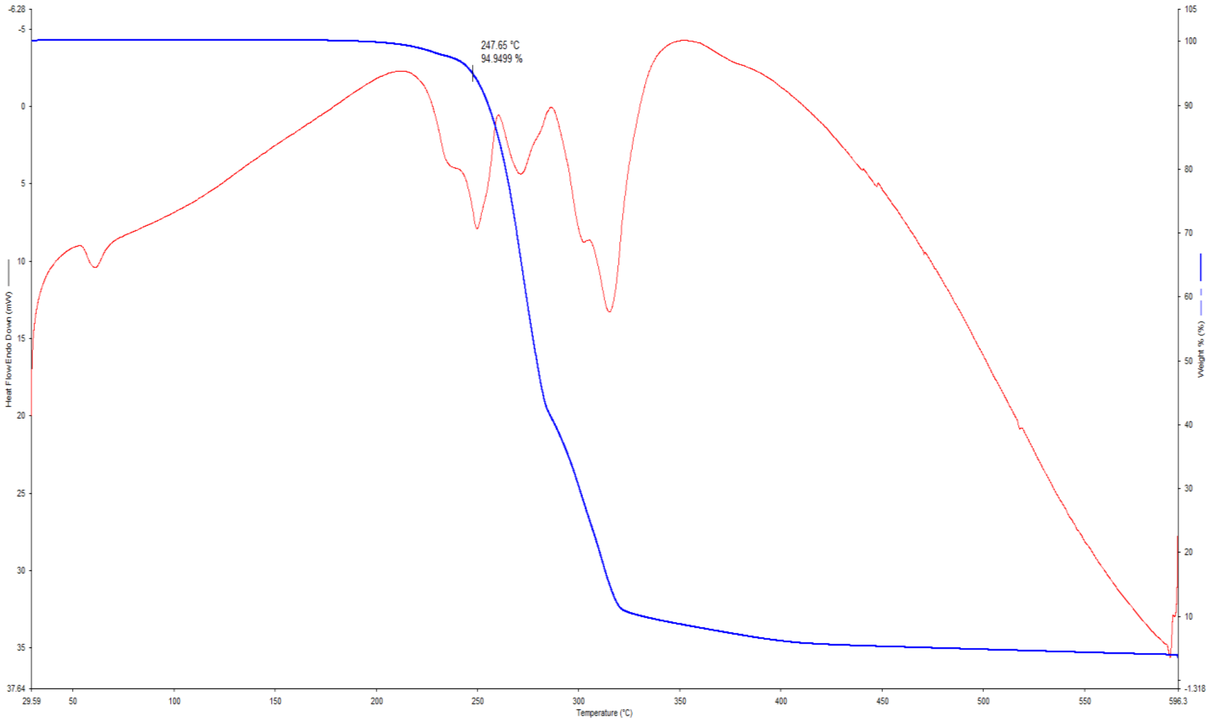

FIGURE S7: TGA of 11; Poly(1,4-pentylene furanoate)

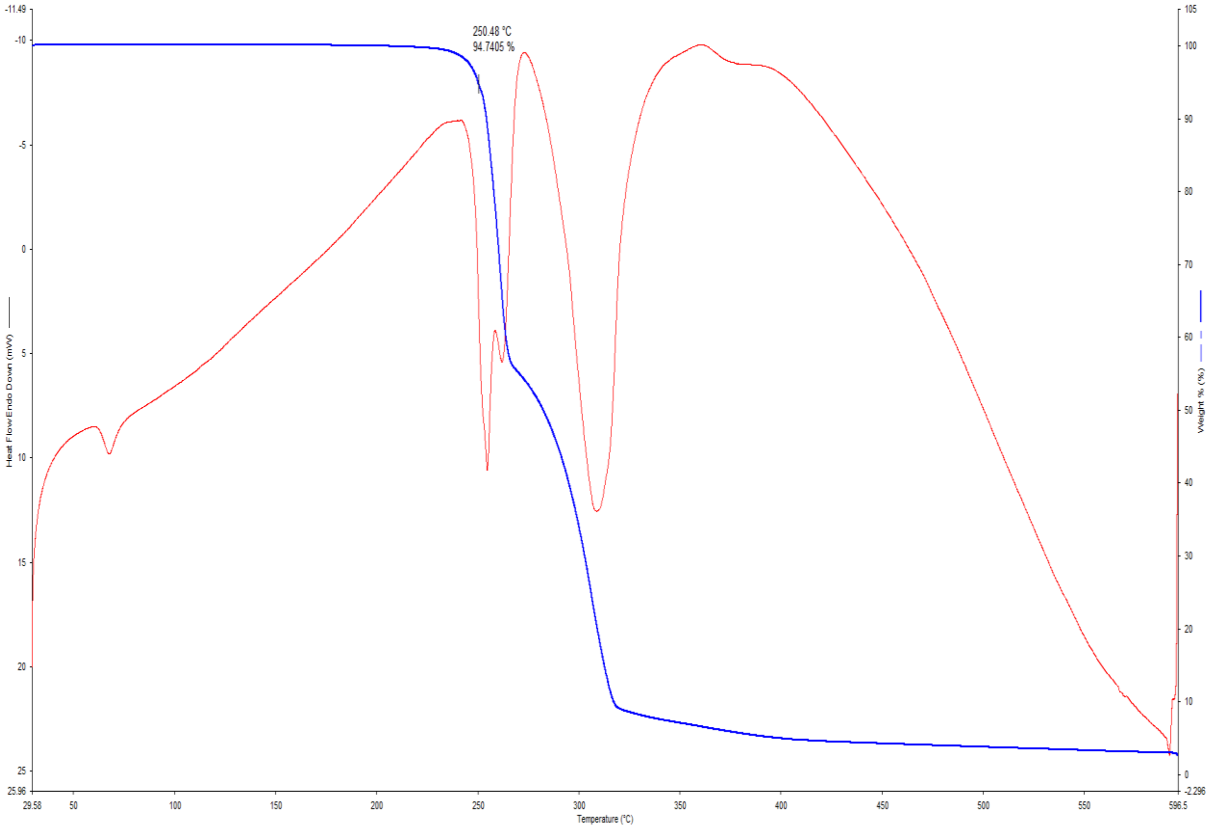

FIGURE S8: TGA of 12; Poly(2,5-hexylene furanoate)

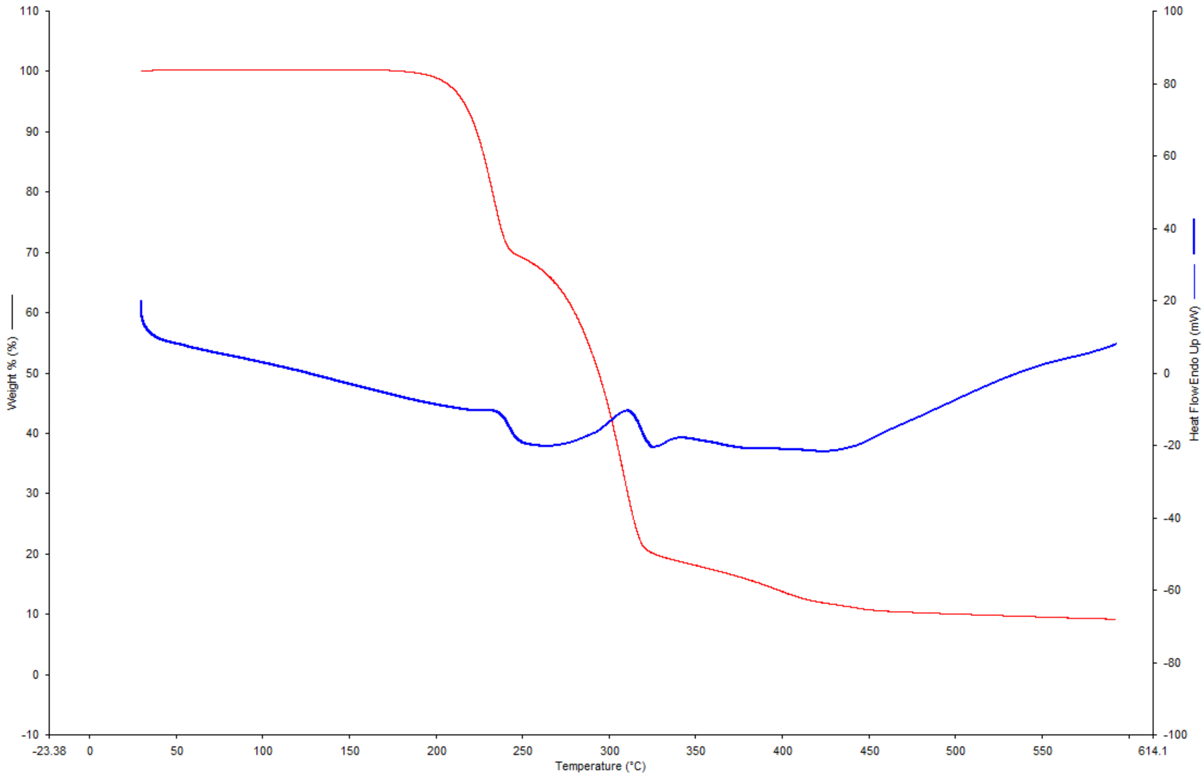

FIGURE S9: TGA of 13; Poly(2*R*,5*R*-hexylene furanoate)

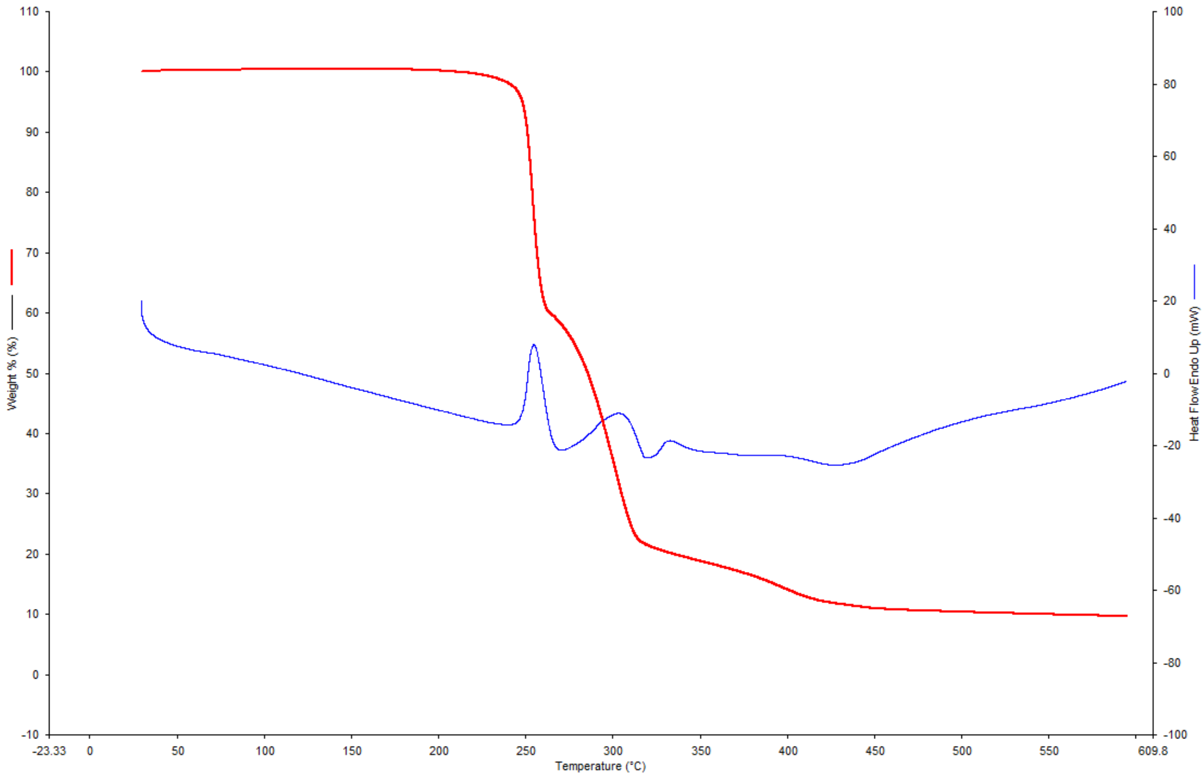

FIGURE S10: TGA of 14; Poly(2*S*,5*S*-hexylene furanoate)

5.2 Differential scanning calorimetry (DSC)

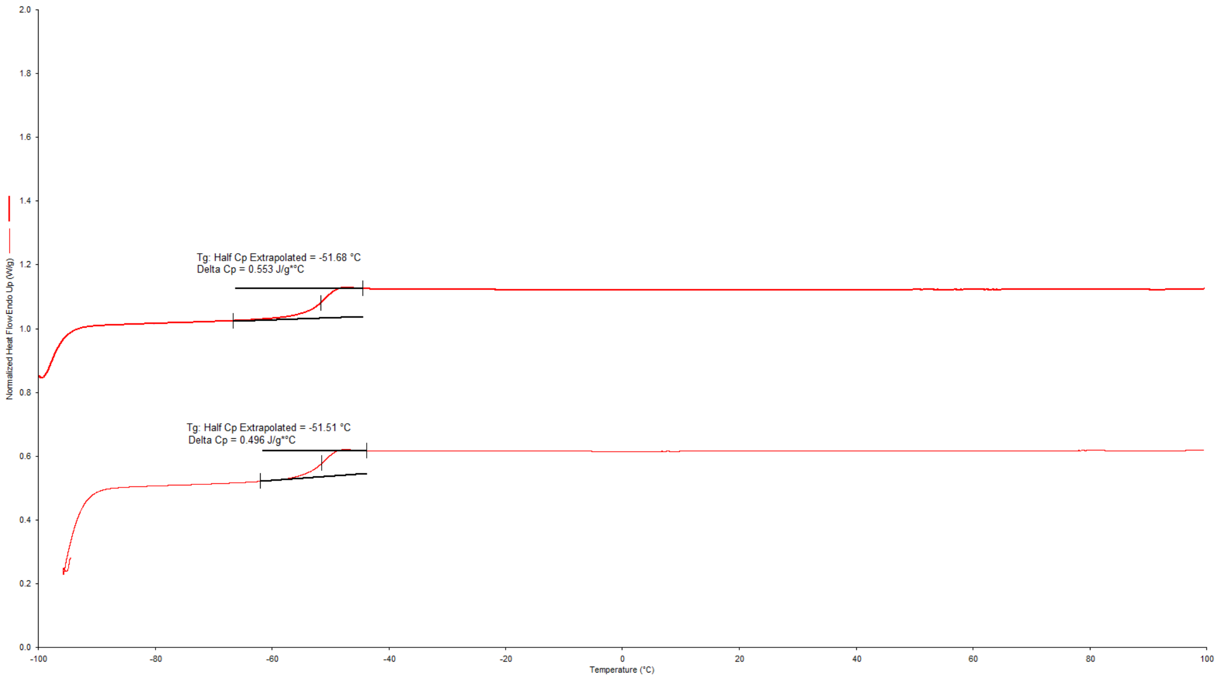

FIGURE S11: DSC of 2; Poly(1,4-pentylene adipate)

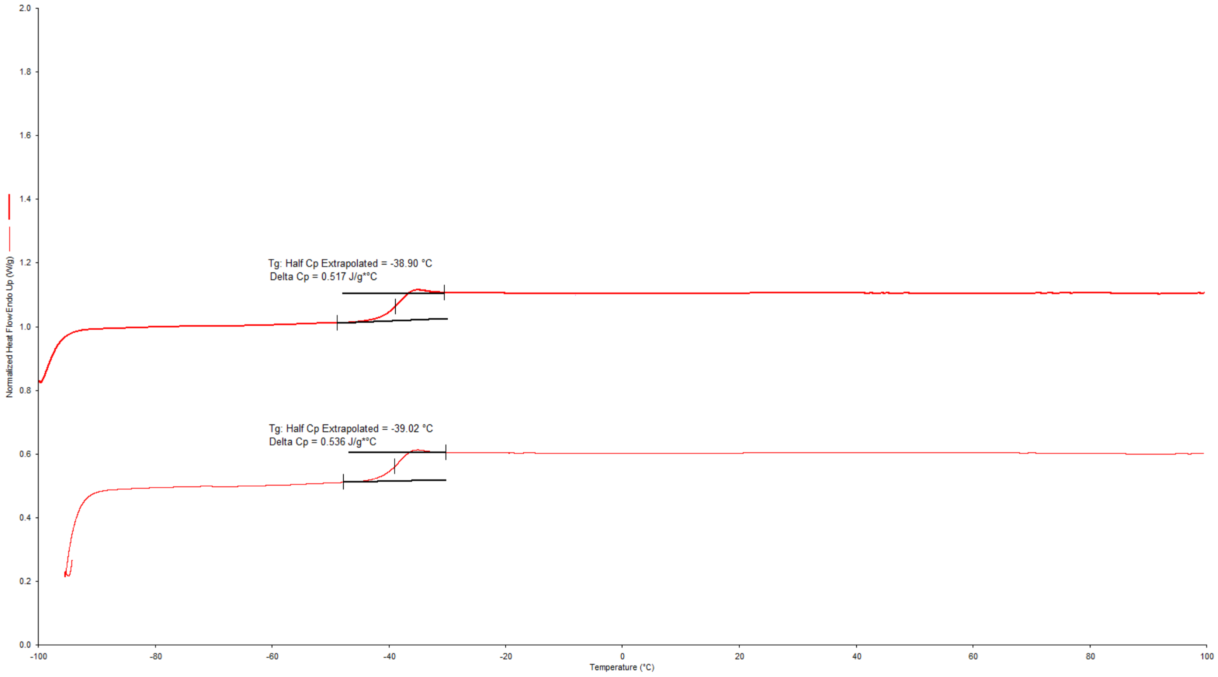

FIGURE S12: DSC of 3; Poly(2,5-hexylene adipate)

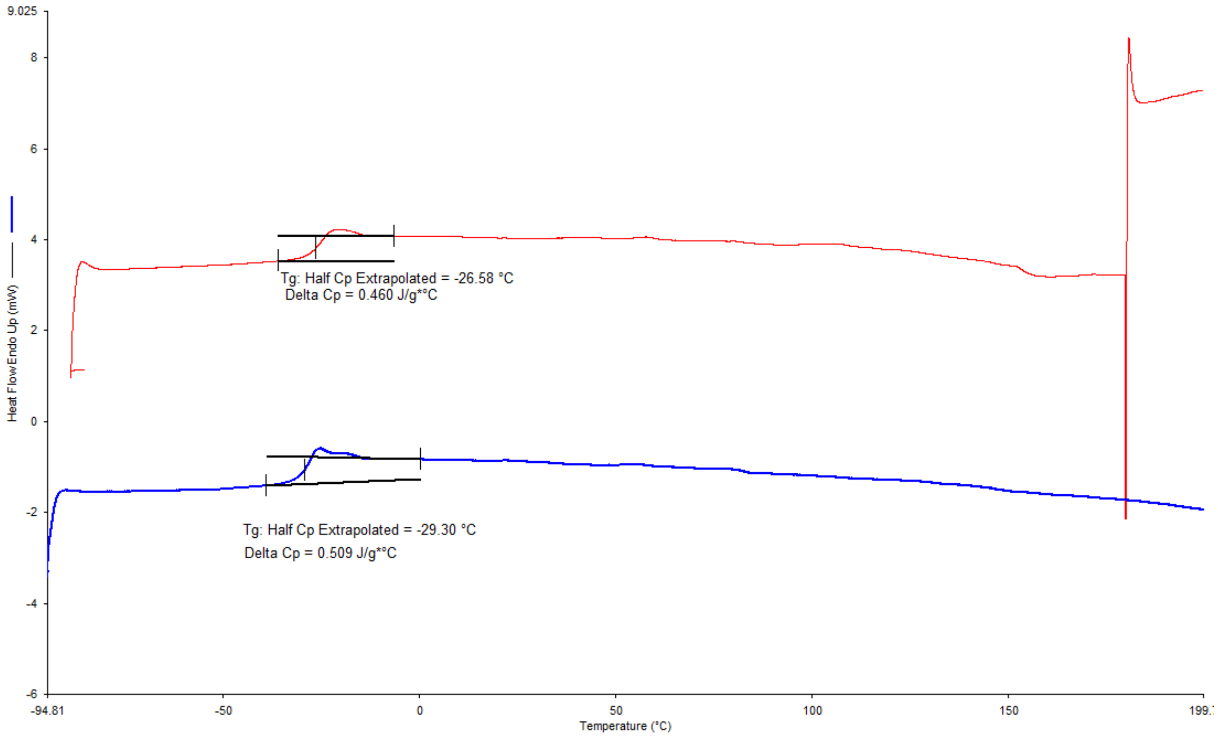

FIGURE S13: DSC of 5; Poly(1,4-pentylene succinate)

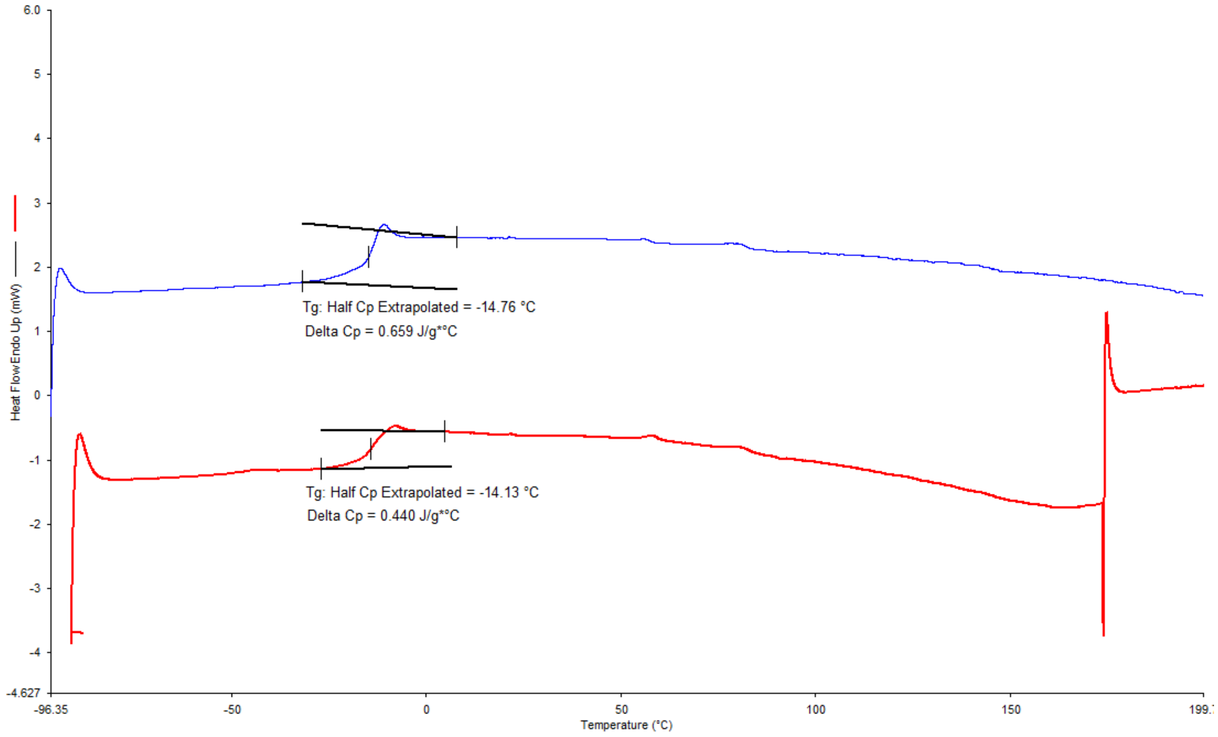

FIGURE S14: DSC of 6; Poly(2,5-hexylene succinate)

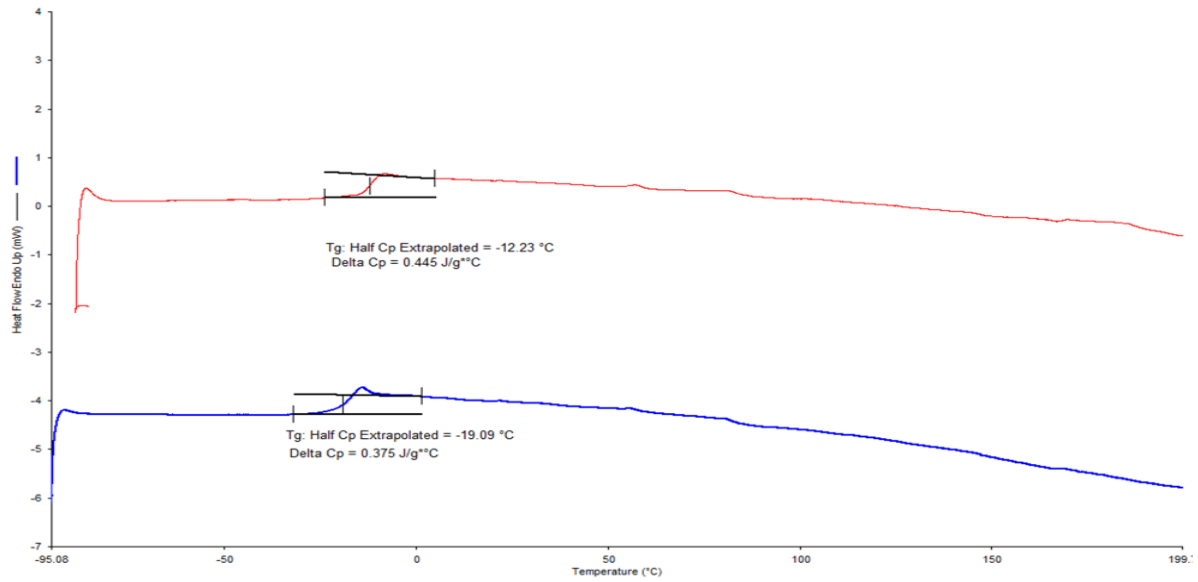

FIGURE S15A: DSC of 7; Poly(2*R*,5*R*-hexylene succinate) (before crystallization)

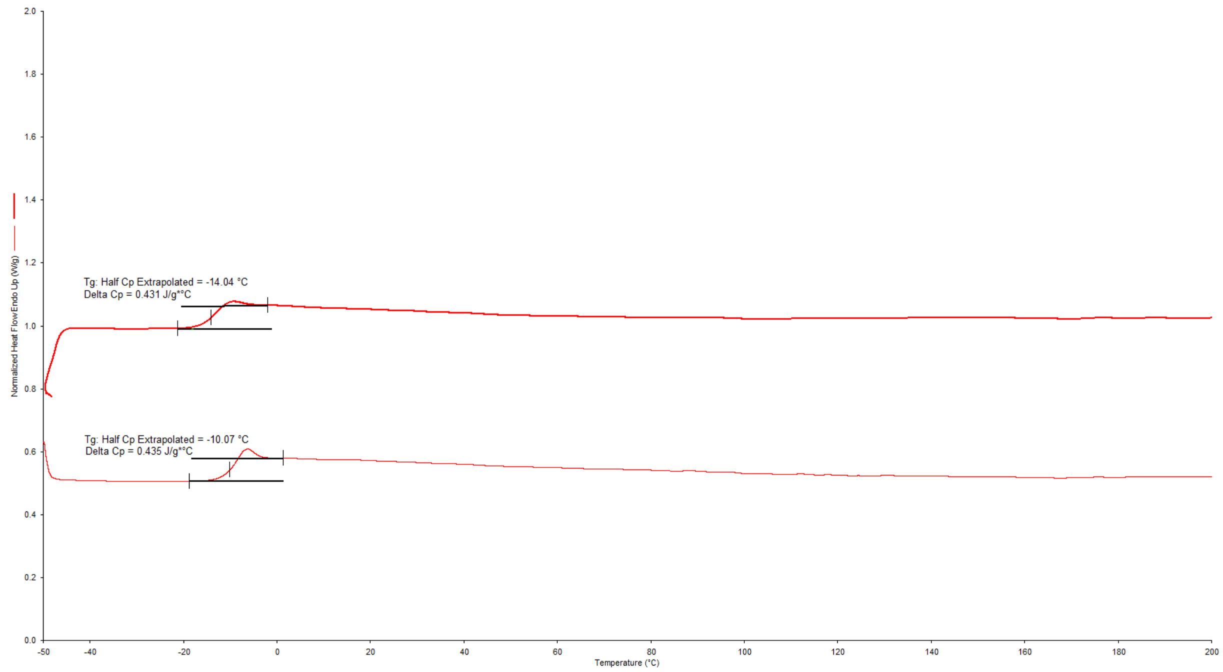

FIGURE S15B: DSC of 7; Poly(2*R*,5*R*-hexylene succinate) (after crystallization)

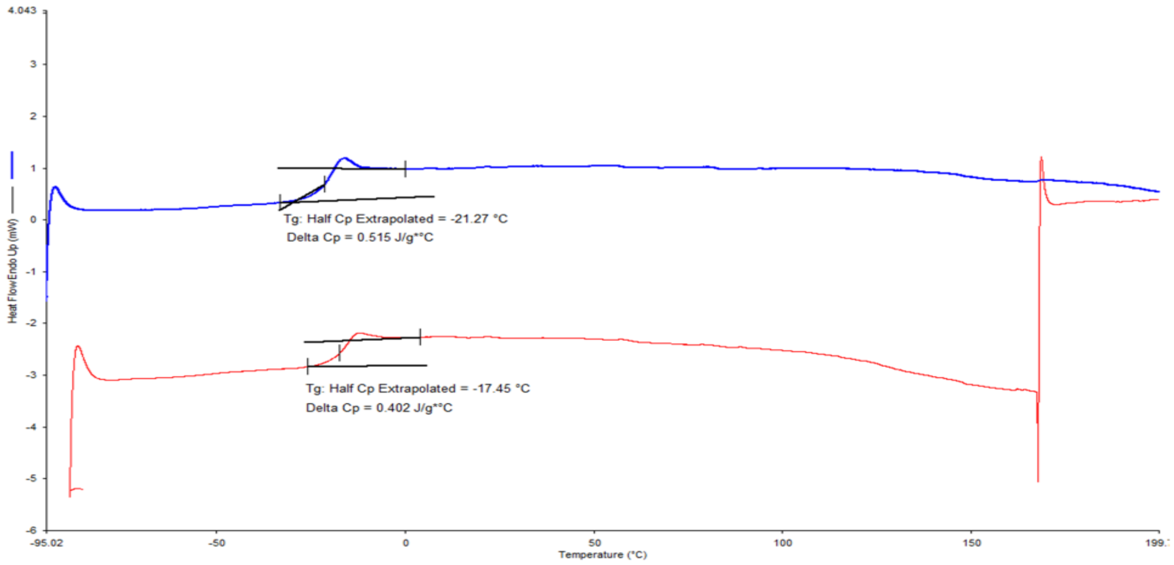

FIGURE S16A: DSC of 8; Poly(2*S*,5*S*-hexylene succinate) (before crystallization)

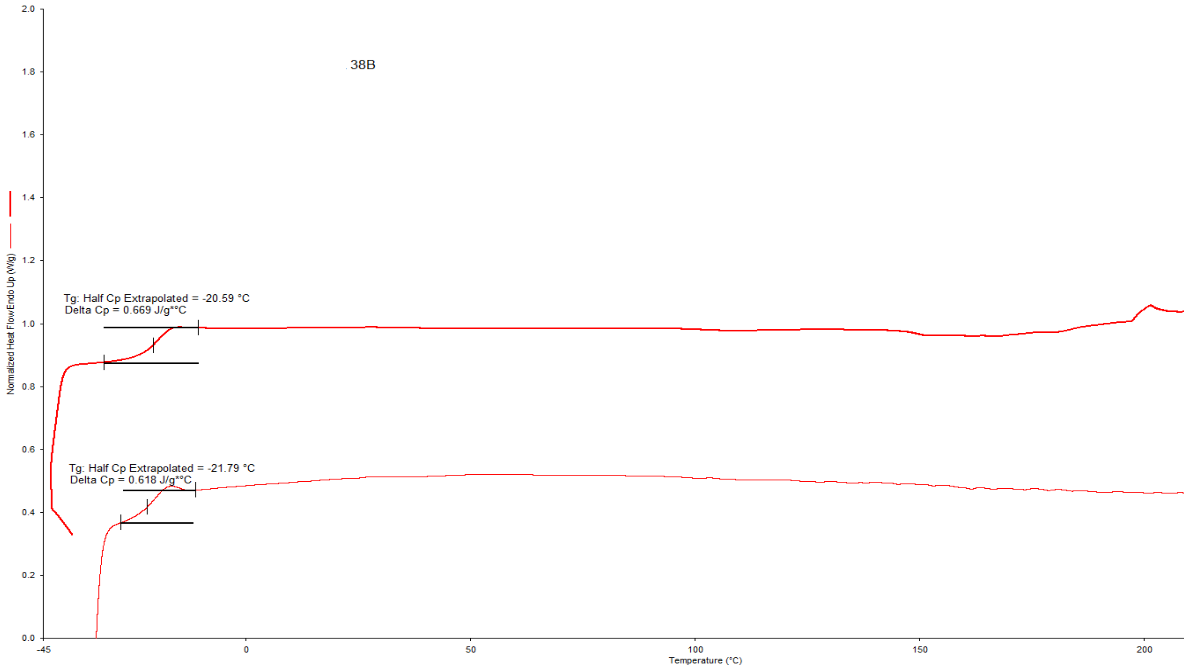

FIGURE S16B: DSC of 8; Poly(2*S*,5*S*-hexylene succinate) (after crystallization)

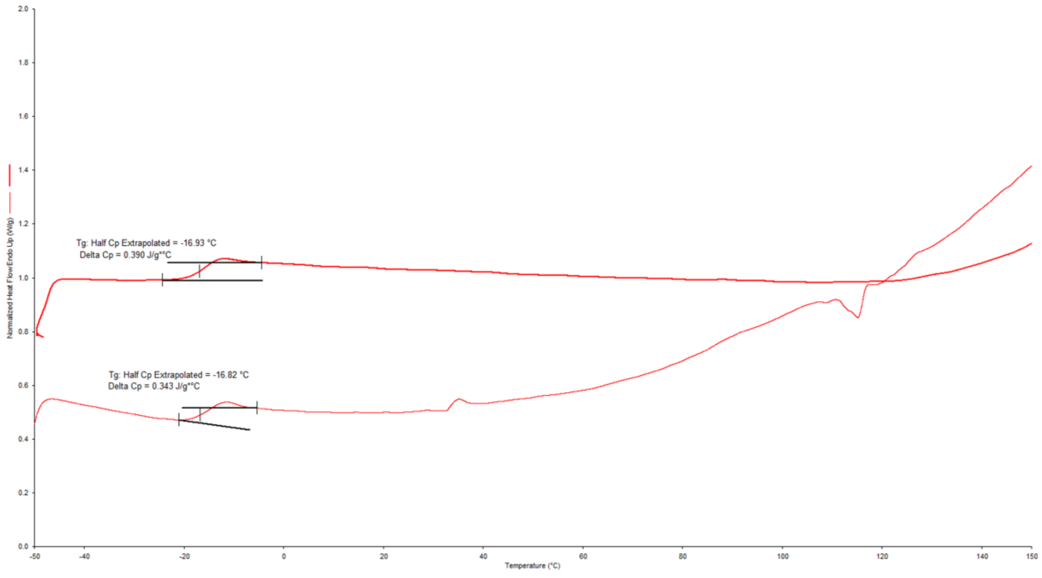

FIGURE S17A: DSC of 9; 1:1 Stereocomplex Poly(2*S*,5*S*-hexylene succinate): Poly(2*S*,5*S*-hexylene succinate)

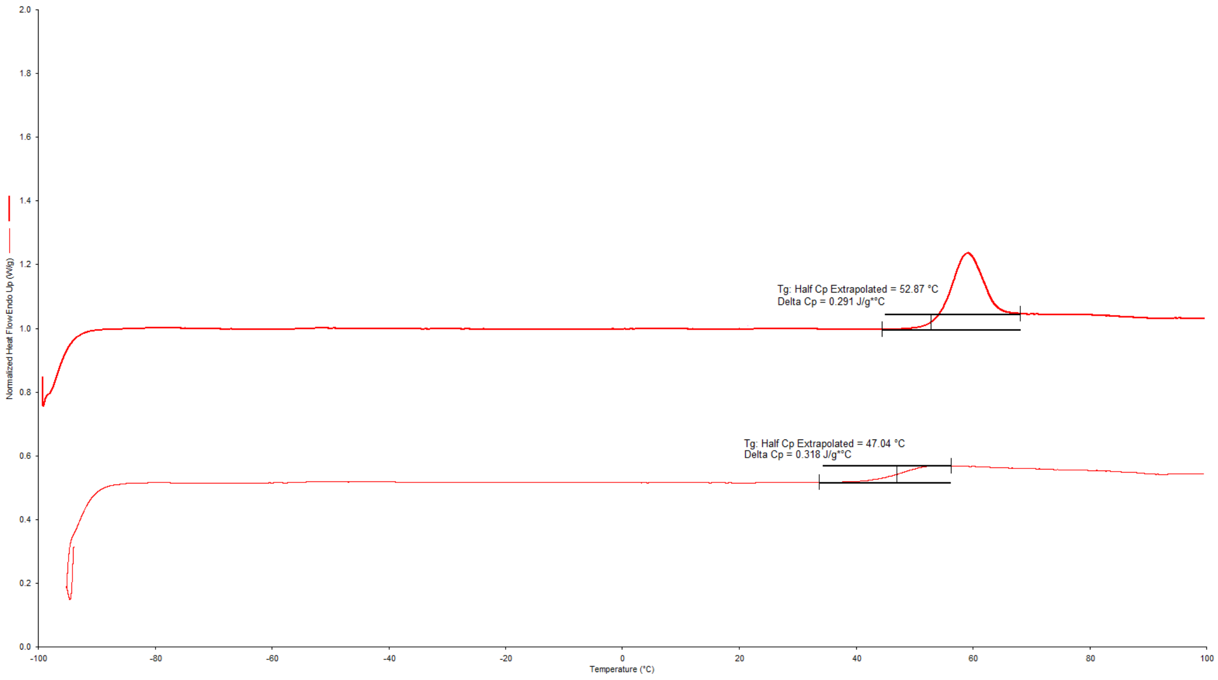

FIGURE S18: DSC of 11; Poly(1,4-pentylene furanoate)

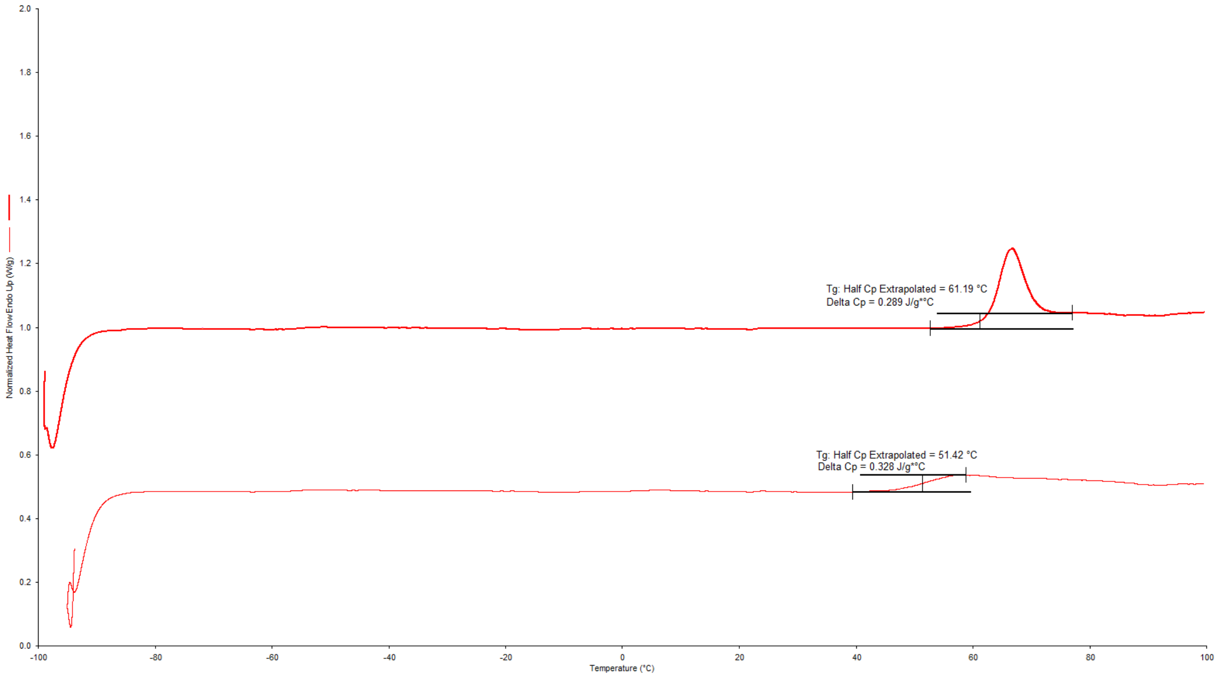

FIGURE S19: DSC of 12; Poly(2,5-hexylene furanoate)

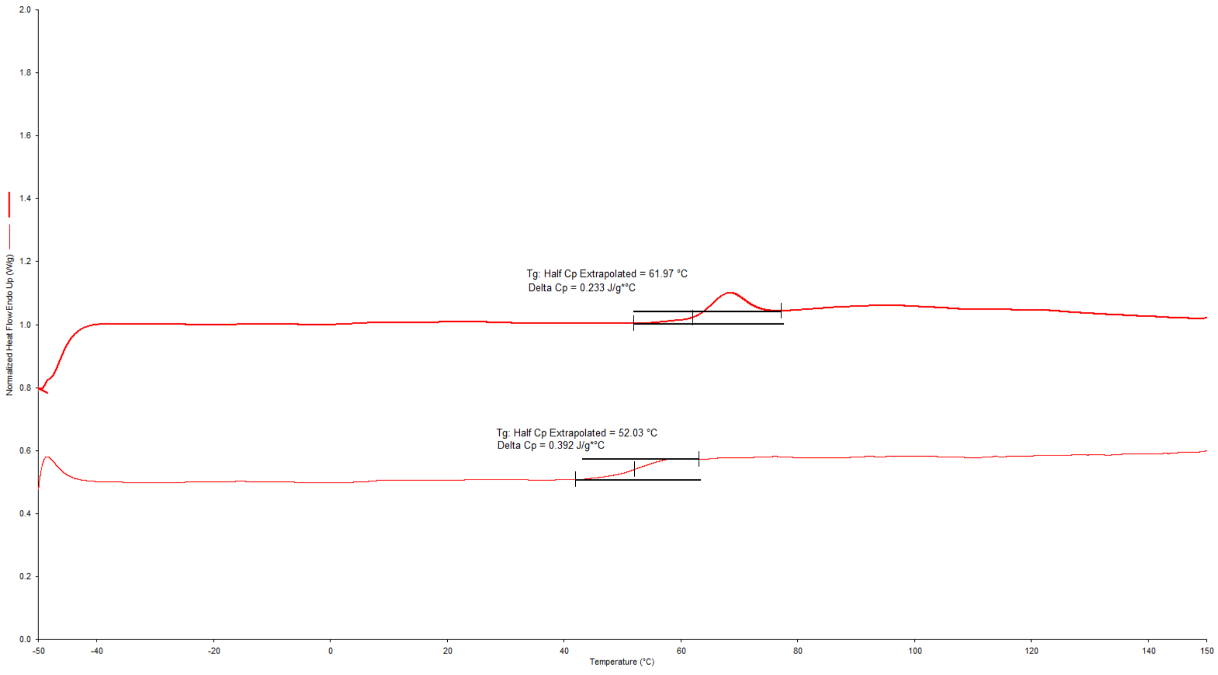

FIGURE S20: DSC of 13; Poly(2*R*,5*R*-hexylene furanoate)

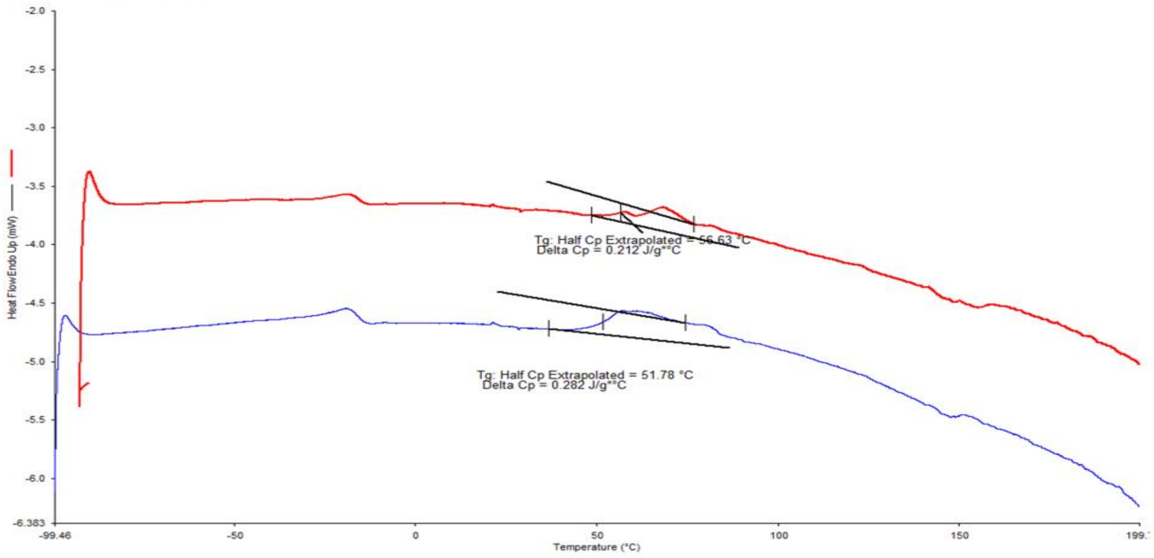

FIGURE S21: DSC of 14; Poly(2*S*,5*S*-hexylene furanoate)

5.3 Matrix assisted laser desorption time-of-flight mass spectrometry (MALDI TOF-MS)

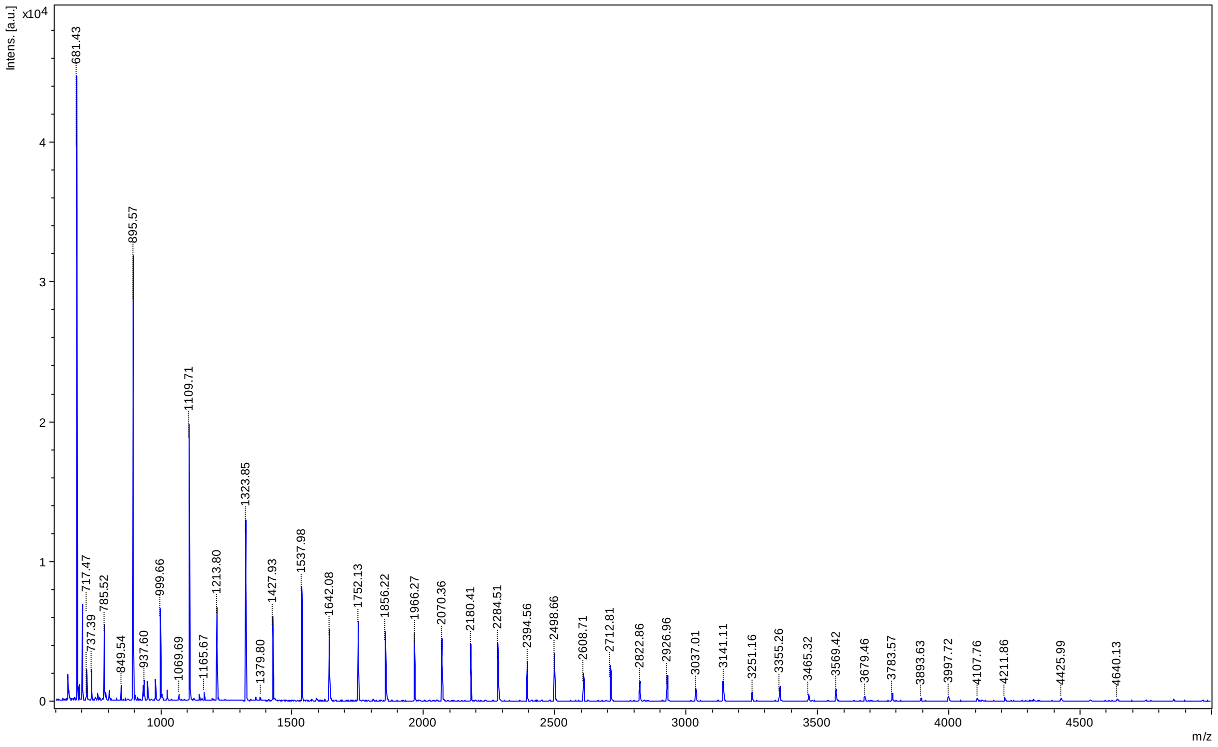

FIGURE S22: MALDI TOF-MS of 2; Poly(1,4-pentylene adipate)

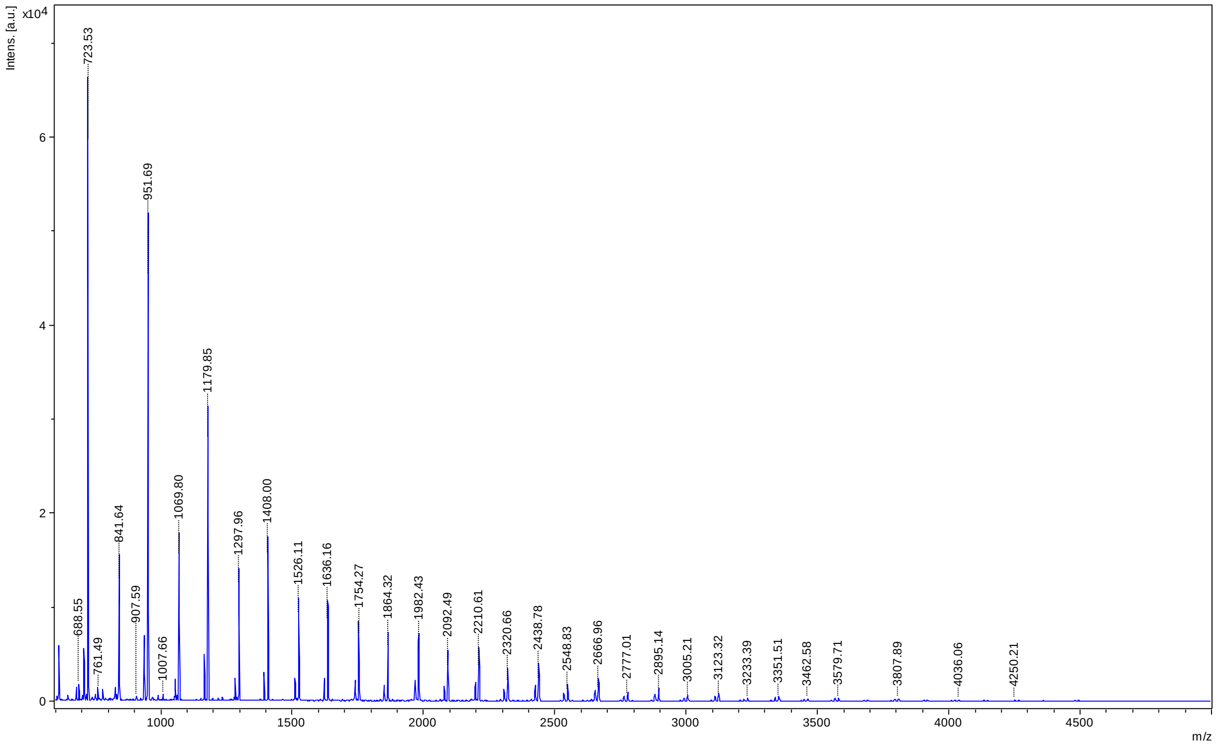

FIGURE S23: MALDI TOF-MS of 3; Poly(2,5-hexylene adipate)

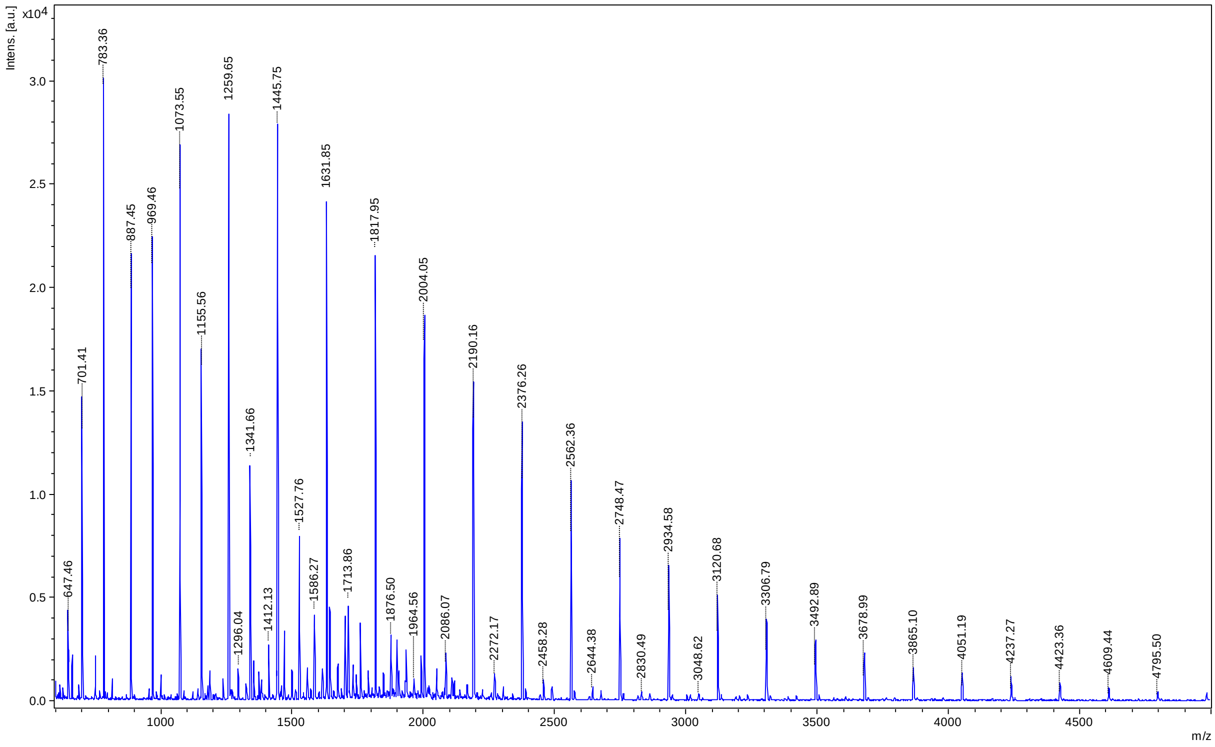

FIGURE S24: MALDI TOF-MS of 5; Poly(1,4-pentylene succinate)

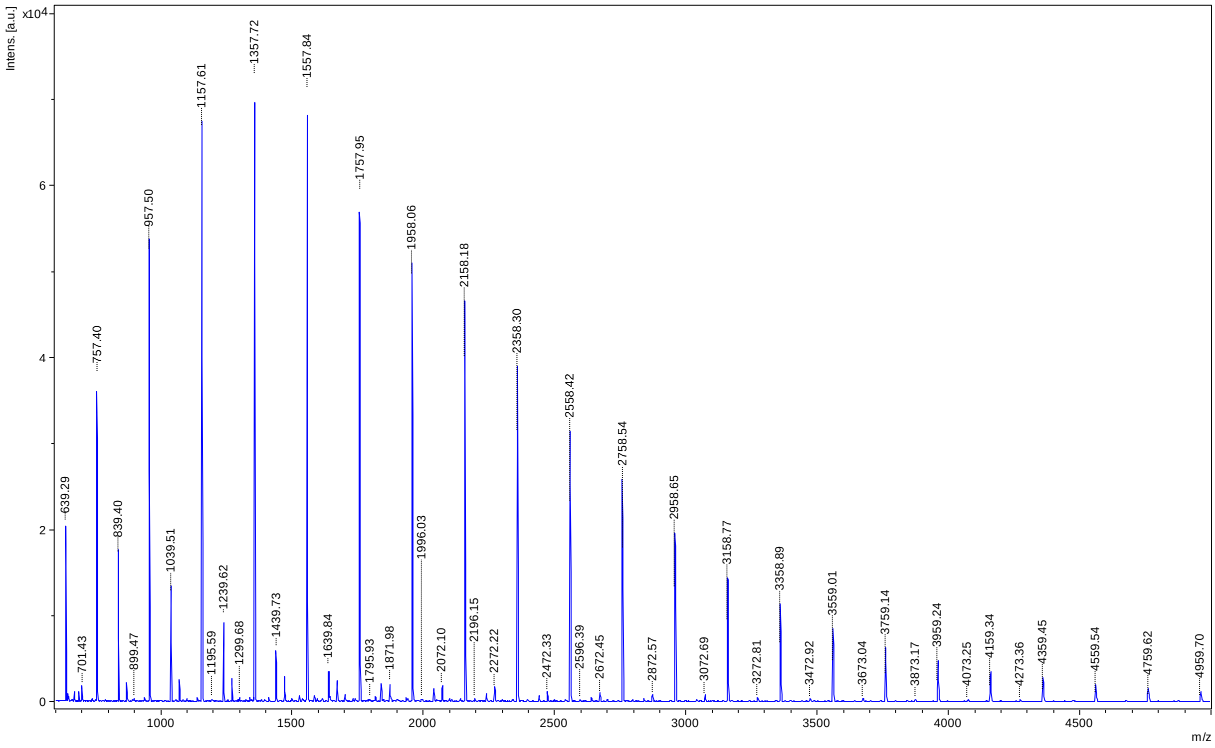

FIGURE S25: MALDI TOF-MS of 6; Poly(2,5-hexylene succinate)

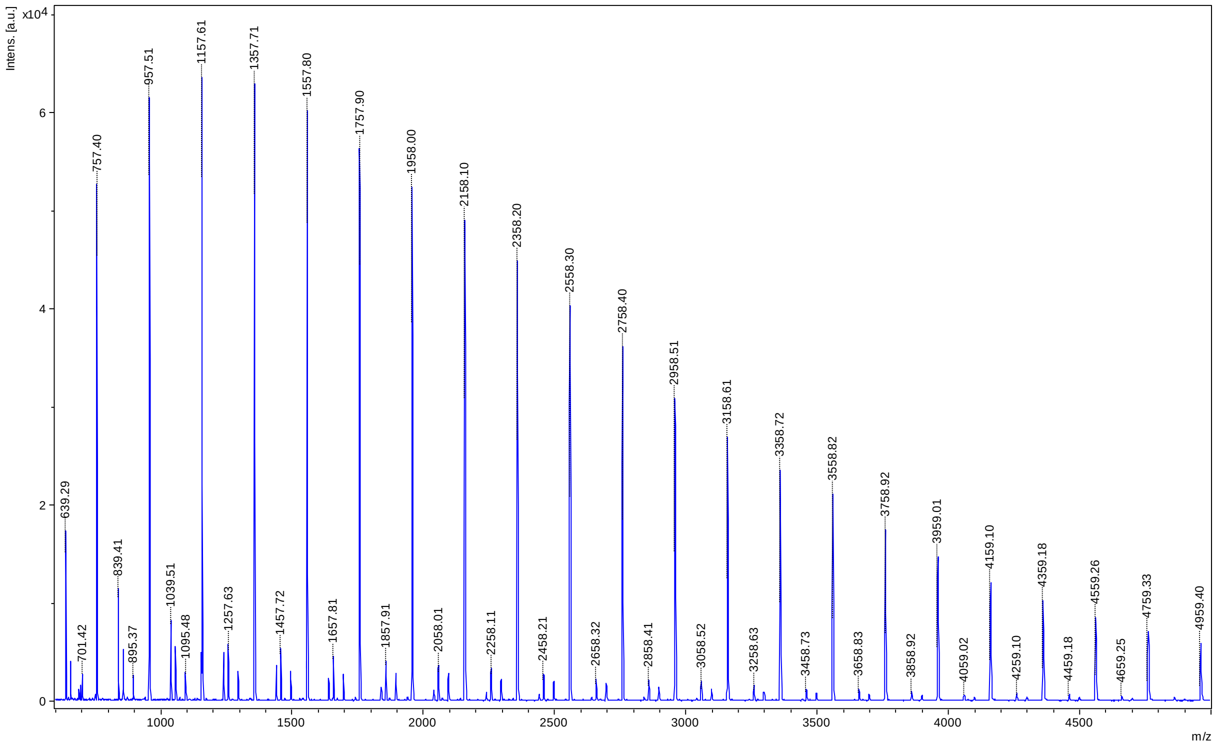

FIGURE S26: MALDI TOF-MS of 7; Poly(2*R*,5*R*-hexylene succinate)

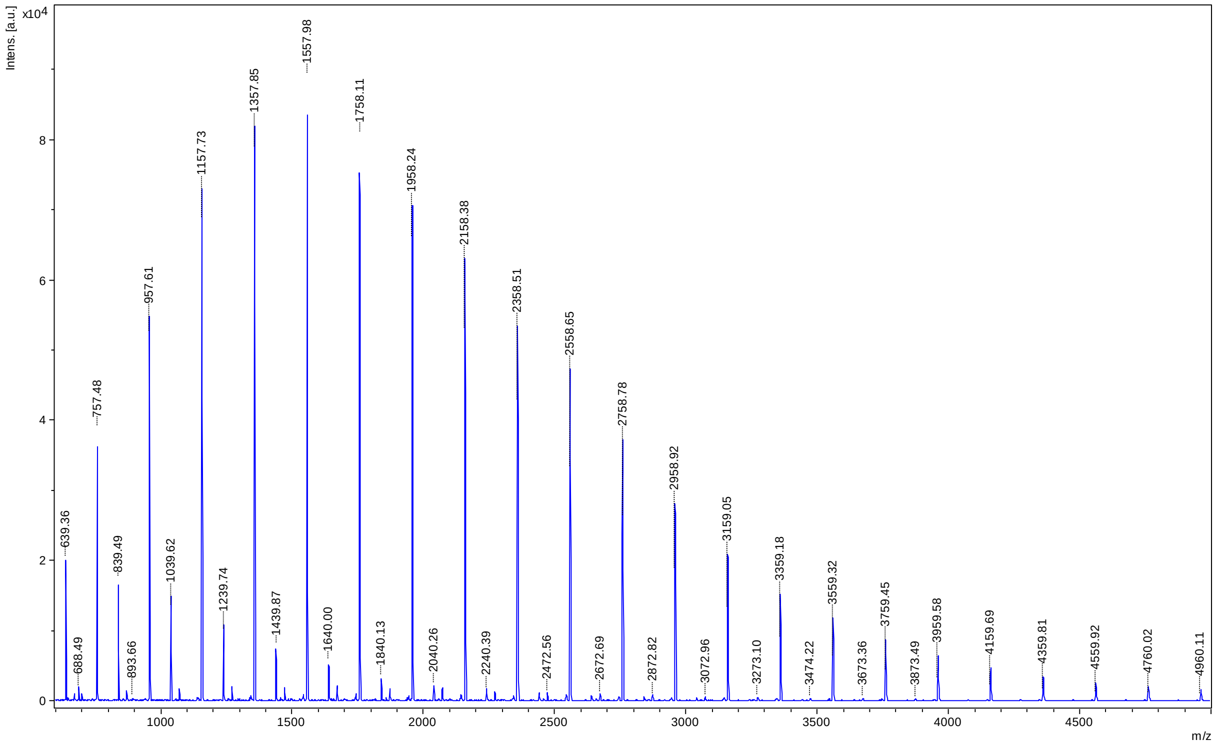

FIGURE S27: MALDI TOF-MS of 8; Poly(2*S*,5*S*-hexylene succinate)

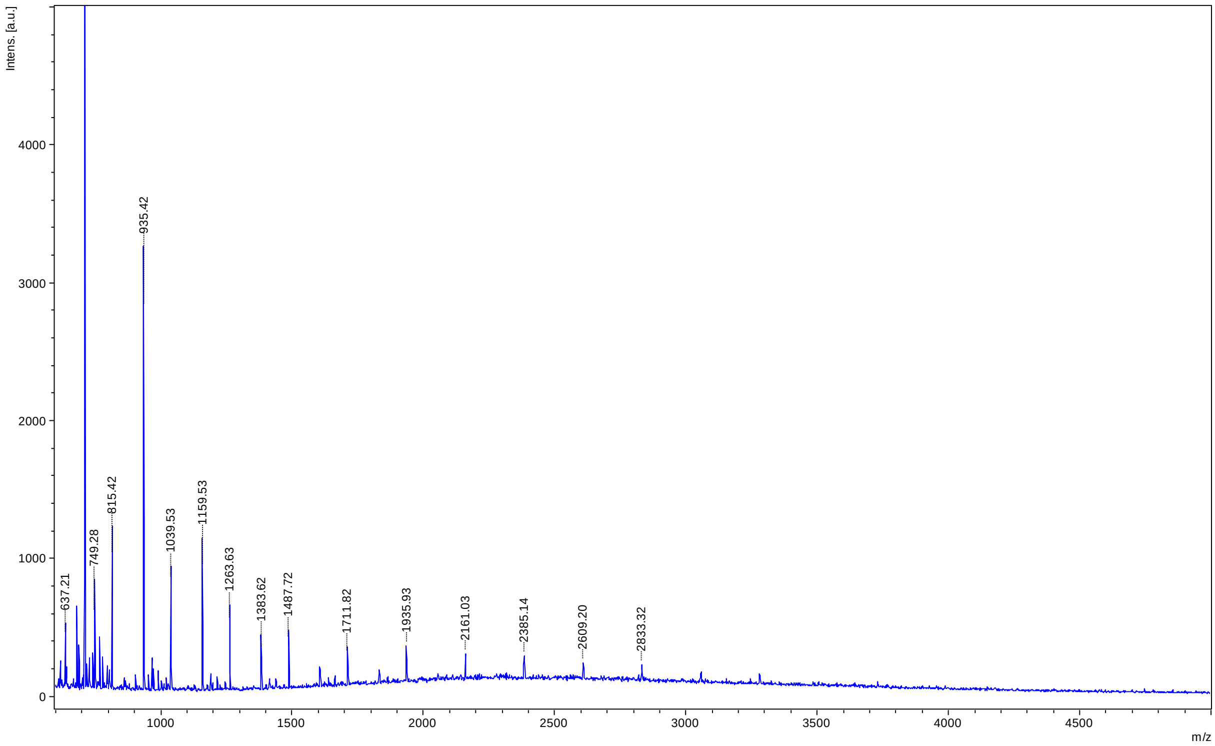

FIGURE S28: MALDI TOF-MS of 11; Poly(1,4-pentylene furanoate)

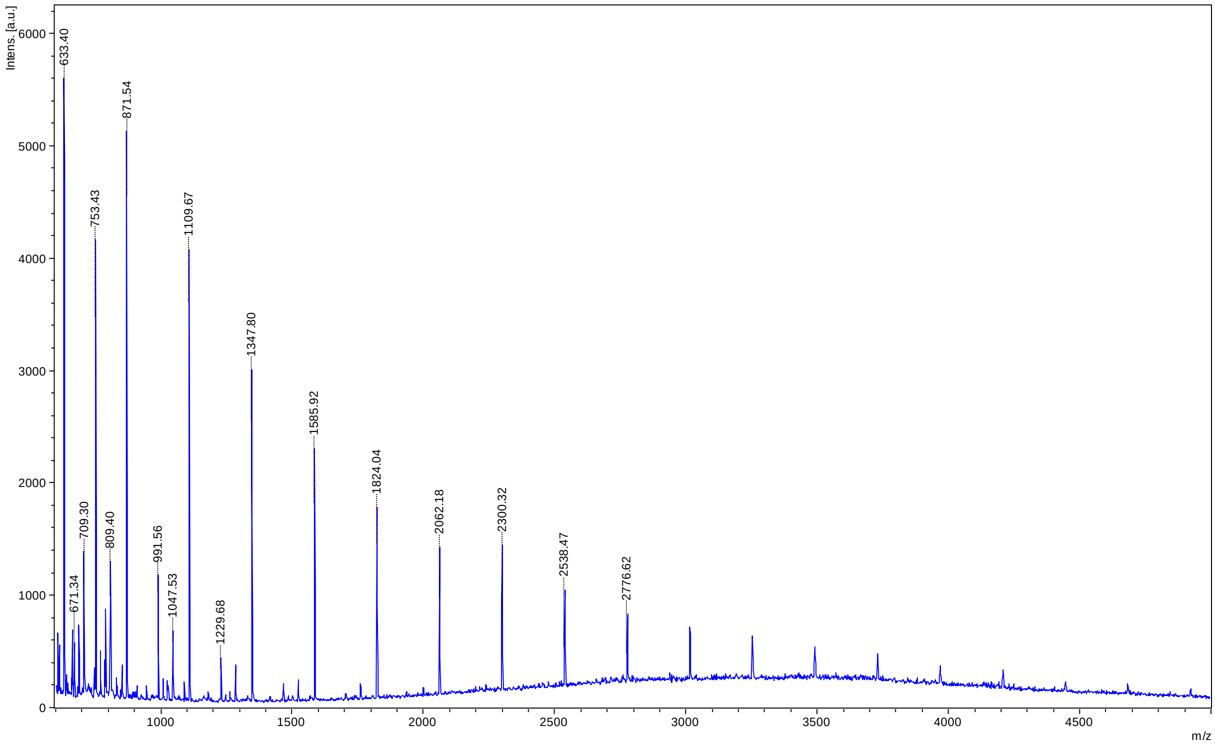

FIGURE S29: MALDI TOF-MS of 12; Poly(2,5-hexylene furanoate)

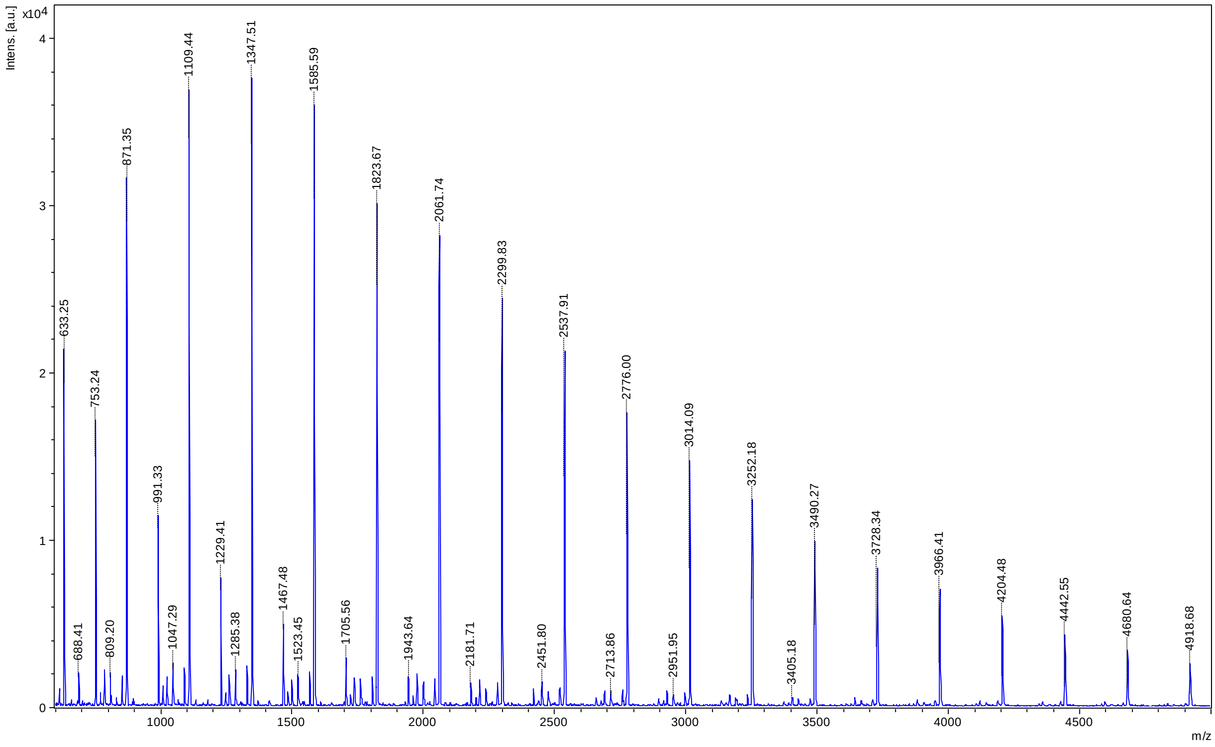

FIGURE S30: MALDI TOF-MS of 13; Poly(2*R*,5*R*-hexylene furanoate)

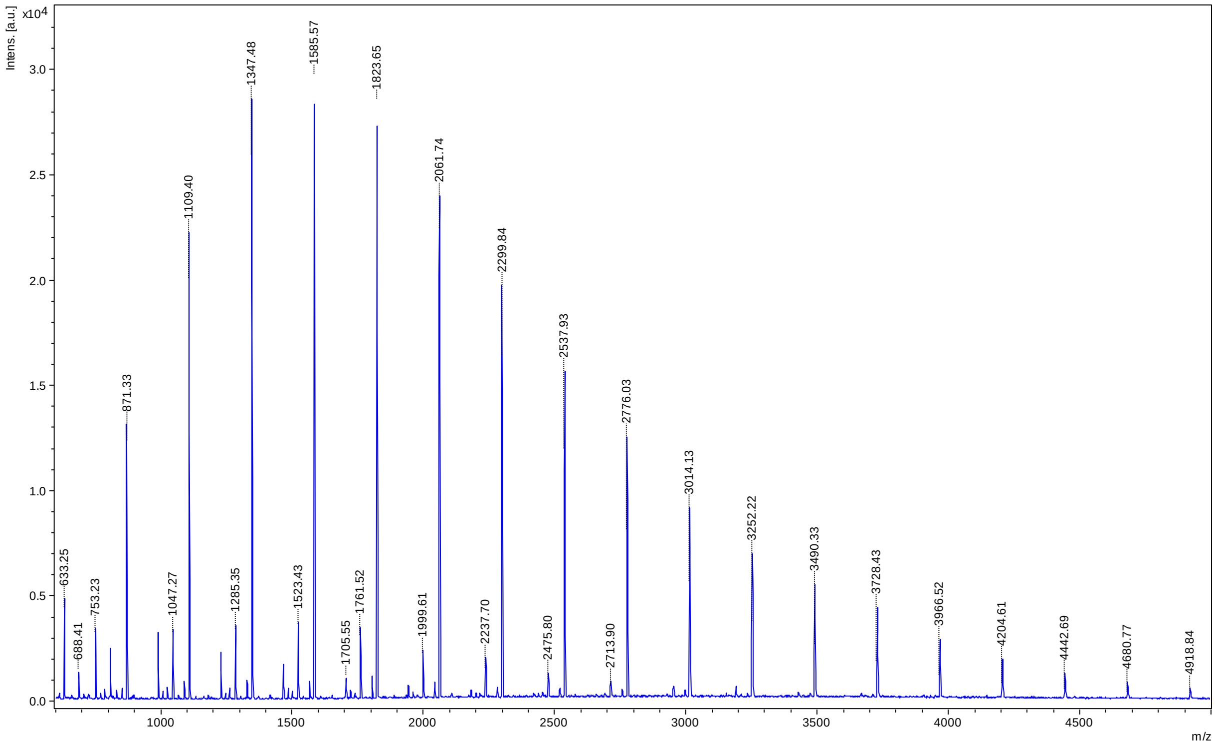

FIGURE S31: MALDI TOF-MS of 14; Poly(2*S*,5*S*-hexylene furanoate)

6. References

1. R. J. I. Knoop, W. Vogelzang, J. van Haveren, D. S. van Es. *J. Polym. Sci., Part A: Polym. Chem.* **2013**, *51*, 4191-4199.
2. F. van der Klis, J. van Haveren, D. S. van Es, J. H. Bitter. *ChemSusChem* **2017**, *10*, 1460-1468.
3. Á. Cruz-Izquierdo, L. A. M. van den Broek, J. L. Serra, M. J. Llama, C. G. Boeriu. *Pure Appl. Chem.* **2015**, *87*, 59-69.
4. S. Elangovan, B. Wendt, C. Topf, S. Bachmann, M. Scalone, A. Spannenberg, H. Jiao, W. Baumann, K. Junge, M. Beller. *Adv. Synth. Catal.* **2016**, *358*, 820-825.
5. K. Moriya, M. Simon, R. Mose, K. Karaghiosoff, P. Knochel. *Angew. Chem. Int. Ed.* **2015**, *54*, 10963-10967.
